# Post-Turing tissue pattern formation: Advent of mechanochemistry

**DOI:** 10.1371/journal.pcbi.1006259

**Published:** 2018-07-03

**Authors:** Felix Brinkmann, Moritz Mercker, Thomas Richter, Anna Marciniak-Czochra

**Affiliations:** 1 Institute of Applied Mathematics, BioQuant and Interdisciplinary Center of Scientific Computing (IWR), Heidelberg University, Heidelberg, Germany; 2 Magdeburg University, Institute for Analysis and Numerics, Magdeburg, Germany; Oxford, UNITED KINGDOM

## Abstract

Chemical and mechanical pattern formation is fundamental during embryogenesis and tissue development. Yet, the underlying molecular and cellular mechanisms are still elusive in many cases. Most current theories assume that tissue development is driven by chemical processes: either as a sequence of chemical patterns each depending on the previous one, or by patterns spontaneously arising from specific chemical interactions (such as “Turing-patterns”). Within both theories, mechanical patterns are usually regarded as passive by-products of chemical pre-patters. However, several experiments question these theories, and an increasing number of studies shows that tissue mechanics can actively influence chemical patterns during development. In this study, we thus focus on the interplay between chemical and mechanical processes during tissue development. On one hand, based on recent experimental data, we develop new mechanochemical simulation models of evolving tissues, in which the full 3D representation of the tissue appears to be critical for obtaining a realistic mechanochemical behaviour. The presented modelling approach is flexible and numerically studied using state of the art finite element methods. Thus, it may serve as a basis to combine simulations with new experimental methods in tissue development. On the other hand, we apply the developed approach and demonstrate that even simple interactions between tissue mechanics and chemistry spontaneously lead to robust and complex mechanochemical patterns. Especially, we demonstrate that the main contradictions arising in the framework of purely chemical theories are naturally and automatically resolved using the mechanochemical patterning theory.

## Author summary

During embryogenesis, biological tissues gradually increase their complexity by self-organised creation of diverse chemical and mechanical patterns. Detailed mechanisms driving and controlling these patterns are not well understood. Previous theories mostly assume that these patterns are driven by chemical processes. Based on these theories, mechanical patterns are usually considered being mainly determined by chemical pre-patterns. However, experimental evidence for these theories is sparse, and several inconsistencies have been discovered. Furthermore, an increasing amount of data shows that tissue mechanics plays an important role in pattern formation. In this study, we present 3D computer simulations of evolving tissues to investigate the capacity of mechanochemical interactions for pattern formation. We show that even simple interactions between tissue mechanics and tissue chemistry spontaneously lead to robust chemical and mechanical pattern formation. We additionally demonstrate that main contradictions arising in the framework of purely chemical theories are naturally and automatically resolved using the mechanochemical patterning theory. The presented modelling approach can be used to combine simulations with recent experimental developments, to help unravel one of the big mysteries in biology: The mechanisms of self-organised pattern formation during embryogenesis.

## Introduction

During embryogenesis or tissue development, various chemical and mechanical patterns emerge in a self-organised way based on relatively simple structures, such as a tissue sphere [1]. During the last decades, a main focus in developmental biology was the experimental identification of signalling molecules (“morphogens”) being spatio-temporally associated with certain developmental steps in various model organisms [1, 2]. However, the knowledge about *how* chemical patterns are produced, controlled, and how they interact with mechanical patterns is still very unsatisfactory.

A frequent obstacle to the research on mechanochemical pattern formation is the mechanical aspect, since experimental tools for mechanical tissue modification, molecular markers of mechanical cues and mechanochemical modelling are still in its infancy [3]. Especially, it appears that often the full 3D nature of tissue mechanics has to be considered in experiments and models for obtaining results which can be related to *in vivo* processes [4–10] making the situation even more challenging. Thus, although correlations between biological forms and mechanical phenomena were already pointed out in the seminal work of D’Arcy Thompson [11], mainly pure chemical theories have been predominated hereof during the last century of research in order to explain tissue pattern formation during development.

The first of these theories assumes that embryogenesis is a sequence of successive chemical patterns, where each chemical pattern relies (in other words, depends sensitively) on the previous pattern [12]. A model organism partially fitting this theory is the fruit fly *Drosophila*, in which the orientation of the initial body axis sensitively depends on the maternally inherited Bicoid RNA [13], and later stages are defined by distinct chemical patterns (“gap genes” [14]). However, experimental studies show that embryonic patterns are often robust to removal, addition or redistribution of embryo parts during the preceding patterning stages [1, 15, 16]. Moreover, for an increasing number of biological systems it even appears that patterning does not rely on any pre-pattern, but it may develop in a self-organised way from dissociated and re-aggregated cells [17–21]. This capacity of self-organisation during pattern formation is called “*de novo*” or “spontaneous” or “self-organised” pattern formation and strongly disagrees with the above mentioned theory.

Due to these difficulties, a second, more robust theory of pattern formation has received increasing attention, namely a theory which assumes that pattern formation occurs spontaneously and robustly just by specific interactions among diffusing chemicals (“morphogens”) with possible involvement of the chemical environment. This theory was based on the pioneering work of Alan Turing [22] and its extensions by Gierer, Meinhardt, and Murray [23–25], and others. In contrast to previous studies on biological patterning [11, 12], these new approaches were not restricted to pure description of patterns, but offered the possibility to explain their genesis by *de novo* mechanisms [26]. Originally, such “Turing patterns” are assumed to be driven by the mutual interaction of a slowly-diffusing activator morphogen interacting with a fast-diffusing inhibitor morphogen in a specific nonlinear manner (“short range activation and long range inhibition”) [25]. Later works showed that the long-range inhibition does not necessarily require a diffusing inhibitor, but can also result from the depletion of a substrate that is recruited as a result of self-enhancement of the activator (“activator-depleted substrate mechanism” [23, 27]). However, beside these Turing models, other chemical *de novo* models for pattern formation have been proposed, such as the Swift-Hohenberg equation which requires only one diffusing and reacting chemical in order to spontaneously produce patterns [28]. A variety of non-Turing patterns arising in systems coupling one diffusing component with a non-diffusing subsystem has been recently shown in Refs. [29–31].

These purely chemical theories (including the Turing models) are still among the central concepts of developmental biology. However, this theory is not devoid of serious difficulties, such as the following ones:

- after more than 60 years of research, the experimental verification of classical Turing-type morphogens (activator/inhibitor) showing properties proposed by the theory is still very rare: e.g. an appropriate candidate for the long-range inhibitor is still missing in many cases [32, 33];
- diffusion rates as required for Turing-type long-range inhibitors are often at or beyond the limit of measured diffusion rates in biological tissues, especially for patterns appearing on larger tissue scale [34];
- evidence for the Swift-Hohenberg models as well as for the activator-depleted substrate mechanism experimental is sparse; for the latter there exist candidates for subcellular patterns [35, 36] but not for patterns on tissue scale;
- in many developmental processes, dynamic and complex tissue geometries are likely to prevent the establishment of long range inhibitor gradients [37]; and finally,
- the Turing-theory requires highly nonlinear interactions among different types of morphogens in order to produce *de novo* patterns, which makes the underlying assumptions regarding molecular interactions relatively complex [38].

Finally, the two chemical theories we discussed usually assume that mechanical patterns are “blind” end-results of chemical pre-patterns. In contrast, various recent studies show that mechanical patterns are not only passive results of chemical pre-patterns, but can play instead a central role by being actively involved in tissue pattern formation (Ref. [39–43], beyond many others). This agrees well with the observation that mechanical cues can be translated in various ways in order to influence and control chemical patterns, leading to the rapidly evolving research area of mechanotransduction in cell biology [44–48].

Hence, due the experimental observations and disagreements listed above, tissue mechanics is increasingly moving into focus to explain features of pattern formation which have been previously ascribed to diffusing molecules according to the Turing theory. Notably, forces and flows generated by motor proteins or advection have been proposed to significantly increase diffusion rates for long-range inhibition [34]; and different mechanical cues such as curvature, stretch, strain, or compression have been theoretically shown to successfully work as long-range inhibitors in spontaneous pattern formation [3, 49]. Importantly, there is also an increasing experimental support for mechano-chemical interactions as an important driving force in biological patterning. Examples describing, among other, coupling between diffusing morphogens and tissue bending are summarised in Ref. [33, 50]. Finally, also the Swift-Hohenberg equation has been recently linked to mechanical processes, leading to a possible explanation of different biological patterns such as finger prints [51]. However, a general mechanochemical theory for robust pattern formation is still missing.

Although the need for new modelling approaches integrating chemical (morphogen) and mechanical processes during development has been recently stressed [52], models investigating mechanochemical pattern formation are still rare. One of the first seminal works considering the richness and self-organisation of biological growth and forms was the book “On Growth and form” by D’Arcy Thompson in 1917 [11]. However, even its second edition was written before computers made it possible to develop and study more sophisticated models of biological patterning [26]. One of the first attempts to integrate tissue mechanics in pattern formation models has been the papers by Murray and Oster [53, 54]. Here, the interplay between migrating and contracting cells and a deformable elastic surrounding medium (such as an extracellular matrix) can lead to a variety of patterns. The model has been successfully applied to the process of vasculogenesis [55]. A mechanistically related model in which epithelium cells represent the elastic part and actomyosin cross-bridges depict the contractile units has been proposed by Odell *et al.* [56]. Using finite element simulations, they showed that a simple interplay between stretch-induced active contractility and passive propagation of cell stretch can lead to spontaneous gastrulation in tissue spheres. Indeed, recent simulation studies of different mechanochemical models compared to experimental data indicate that the interplay between tissue stretch and morphogens may trigger spontaneous pattern formation in the *Hydra* polyp [40]. Finally, simulation studies demonstrated that mechanical cues other than stretch such as curvature, strain, or stress [3, 49], may drive *de novo* mechanochemical pattern formation.

However, one of the chief simplifications of the above-mentioned approaches is the representation of the 3D tissue body by a 1D curve or a 2D surface. This simplification may cause bias or unrealistic behaviour in both chemical and mechanical processes. On one hand, the neglect of one or more dimensions may lead to appearance of nonexistent diffusion barriers, since additional dimensions may allow the molecules to move around obstacles, which is not possible if these dimensions are not present in the model. On the other hand, tissue deformations and mechanical cues propagate via direct interactions of cells or molecules. These processes are altered if dimensions are neglected. For example, describing the tissue as an infinitely thin deforming surface [40, 49] neglects apico-basal chemical and mechanical gradients, the latter frequently accompanying deformations. Representing the tissue as a 2D cross-section [3, 56] ignores the 2D nature of circumferential chemistry and mechanics. Example is provided by regions with high Gaussian curvature that cannot be described adequately although they may play a critical role for tissue growth and deformations [9, 10]. Several recent experimental works highlight the importance of considering full 3D tissues in order to obtain realistic tissue behaviour [4–6, 8]. In summary, transferring of the above-cited mechanochemical modelling results to reality is possible only to a limited extent.

In his seminal paper, Turing proposed the integration of mechanical aspects in pattern formation, but restricted his own studies to purely chemical processes, since “…*the interdependence of the chemical and mechanical data adds enormously to the difficulty”* [22]. During the last decades, however, modelling and computation approaches integrating mechanical aspects of morphogenesis have reached a sophisticated level (for reviews, cf. Ref. [57, 58]).

In the present study, we thus generalise and extend the existing modelling approaches by introducing a mechanochemical tissue model with the following features:

- tissue is represented by a time-dependent deformable 3D body formulated in the framework of continuum-mechanics;
- the continuous formulation is blended with an explicit description of cell boundaries, the latter among other representing active forces exerting and possibly showing discontinuities at the plasma membrane (“actomyosin cortex”);
- model equations allow an arbitrary coupling between morphogen dynamics and different mechanical cues, such as curvature, strain, or compression and stretch;
- simulations are based on the state of the art finite element library Gascoigne3D [59] in conjunction with the possibility of local mesh control, multigrid methods as well as parallelisation to ensure optimal stability and minimal simulation times.

Since recent experimental efforts to visualise and study tissue mechanics are promising [60–63], the proposed modelling approach may offer a future basis to verify new experimental hypotheses and to motivate experiments, respectively. Close interplay between experimental manipulations and computer simulations will help to further unravel mechanochemical processes leading to robust patterns during tissue development.

To demonstrate the capacity of mechanochemical interactions in *de novo* pattern formation, we additionally use our modelling approach to simulate other feedback loops between morphogen dynamics and mechanical measures. Especially, we show how different, simple interaction rules lead to spontaneous and robust mechanochemical pattern formation.

## Results/Discussion

**Fig 1:**
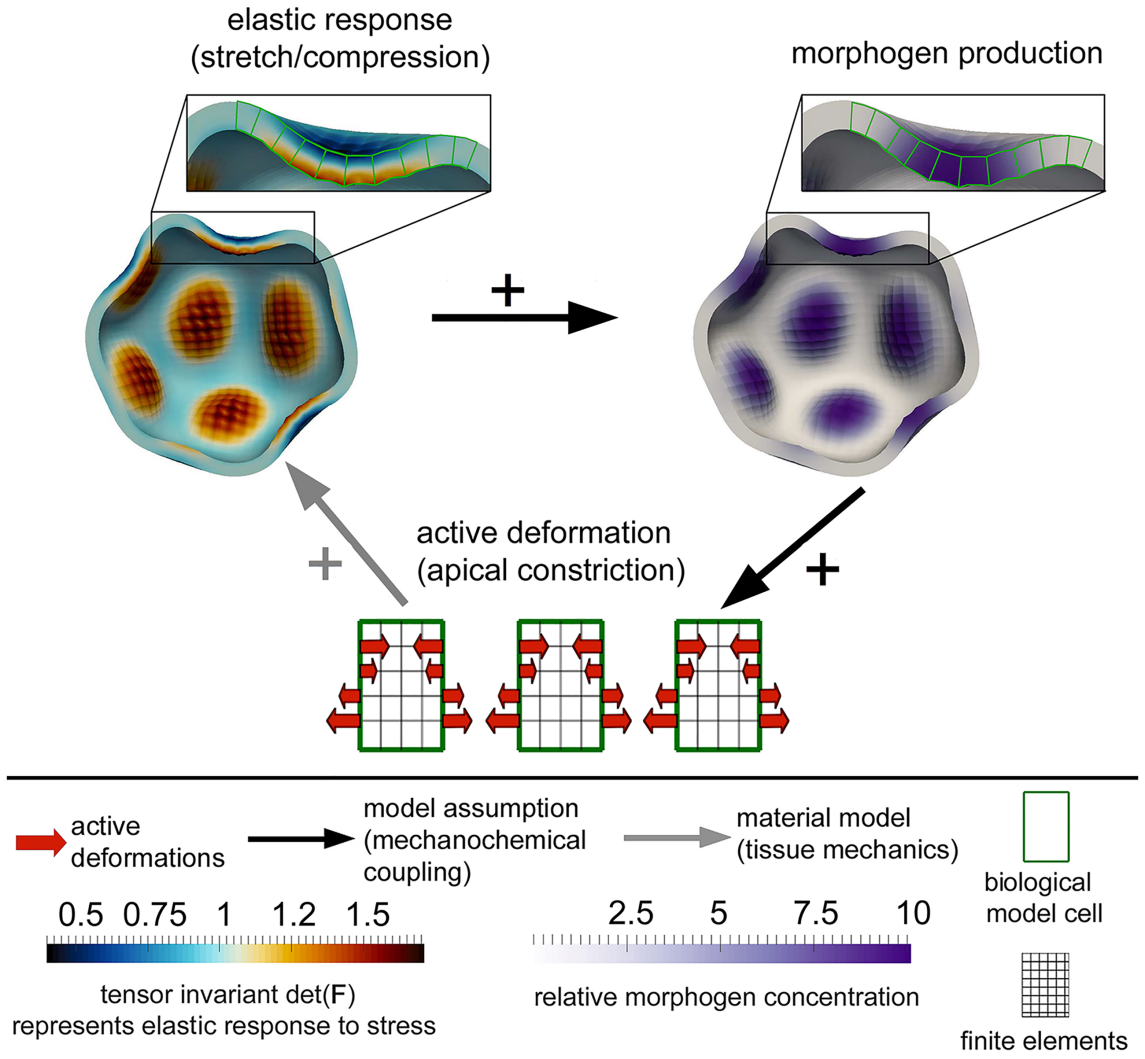
Schematic view of one of the exemplarily investigated feedback-loops between tissue mechanics and morphogen production. Local morphogen levels lead to apical constriction in biological cells, which leads (due to the elastic response of the tissue maintaining continuity) to local stretch, which induces again local morphogen production.

In this work, we combine the most commonly observed interplays between chemistry and tissue mechanics to create a simple feedback loop. Namely, we assume that there exists one morphogen species within the tissue, and that this morphogen locally induces active cell shape changes. Especially, these cell shape changes are assumed to be apical or a basal constrictions (i.e., deforming cells from symmetric to wedge-shaped), since these are frequently appearing deformations during tissue morphogenesis [64–67]. Furthermore, we assume that tissue stretch induces production of the morphogen, which also is a common experimental observation [39, 68]. These two mechanisms lead to a simple positive feedback loop; an example is illustrated in Fig 1.

Especially, mechanochemical feedback loops of this type have the capacity to spontaneously create patterns for the following reason: as soon as the morphogen or the tissue stretch is locally inhomogeneous, both morphogen and tissue curvature locally amplify each other, since morphogen produces cell shape changes which lead to local stretch (due to the elastic material response), which again leads to morphogen production. Thus, a short range activation takes place. The long-range inhibition, in contrast, is mainly constituted by tissue mechanics: As soon as the tissue is locally curved, in oder to maintain continuity, the surroundings of the curved patch have to be curved into the other direction. In the transition zone, however, the curvature and thus the local stretch vanishes and hence no morphogen is produced (c.f., Fig 1).

**Fig 2:**
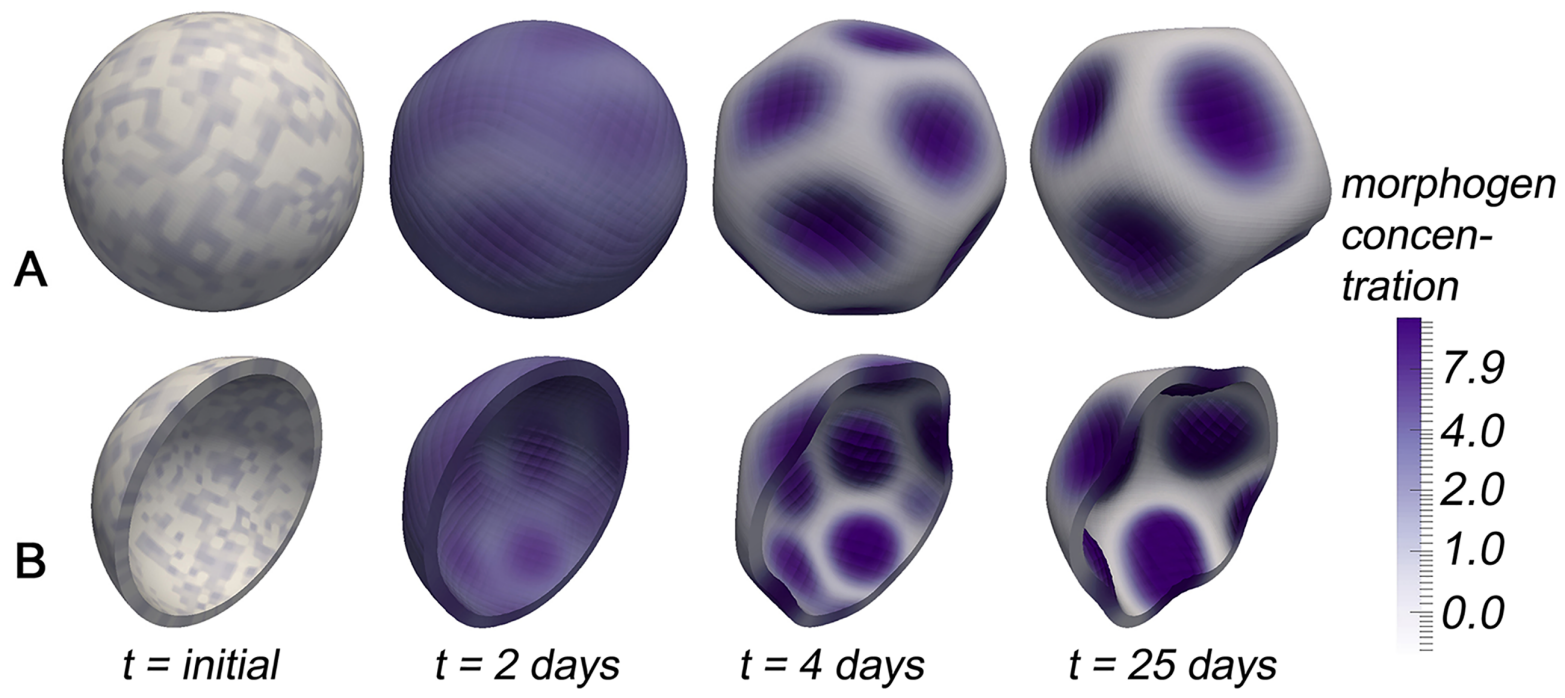
(A)-(B) Simulation snapshots showing spontaneous pattern formation based on a simple mechanochemical feedback loop including basal constriction. In (B), the 3D tissue body has been sliced just for the purpose of a better visualisation. An experimental example showing co-localisation of tissue curvature and morphogen concentration during *Hydra* development can be e.g. found in Ref. [104].

Simulation snapshots for the feedback loop based on basal constriction (i.e., constriction at the end of the cell pointing away from the blastula lumen) are shown in Fig 2. We observe that this simple mechanochemical interplay is sufficient to spontaneously produce regular mechanochemical patterns, where the equilibrium pattern consists of regular morphogen patches coinciding with patches of local tissue curvature. Results appeared to be numerically stationary after *t* ≈ 20 days (referring to the model-time; corresponding to about 85000 numerical time steps), which is a typical order of magnitude for developmental processes. Indeed, co-localisation of high morphogen levels and local tissue curvatures have been described in many organisms and developmental steps, from head formation events in the freshwater polyp *Hydra* [69] through tooth outgrowth in vertebrates [70] and shoot-meristem growth in the plant *Arabidopsis* [71]. Interestingly, for all three processes mentioned above, there are experimental evidences that mechanochemical interactions play an indispensable role during pattern formation [40, 71–74].

Furthermore, the simulated *de novo* equilibrium patterns appear to be very robust against the perturbation of preceding patterning stages, the latter represented by the choice of initial conditions: Regardless if we start with stochastically distributed morphogen levels in biological cells (Fig 2A and 2B) or only one morphogen spot at one side of the tissue sphere (Fig. B in S1 Text) or any other non-homogeneous initial morphogen distribution, we always obtain approximately the same number and size of mechanical and biological patterns. This robustness agrees well with the experimental observation that embryonic patterns are robust to the perturbation of preceding patterning stages [1, 15, 16].

**Fig 3:**
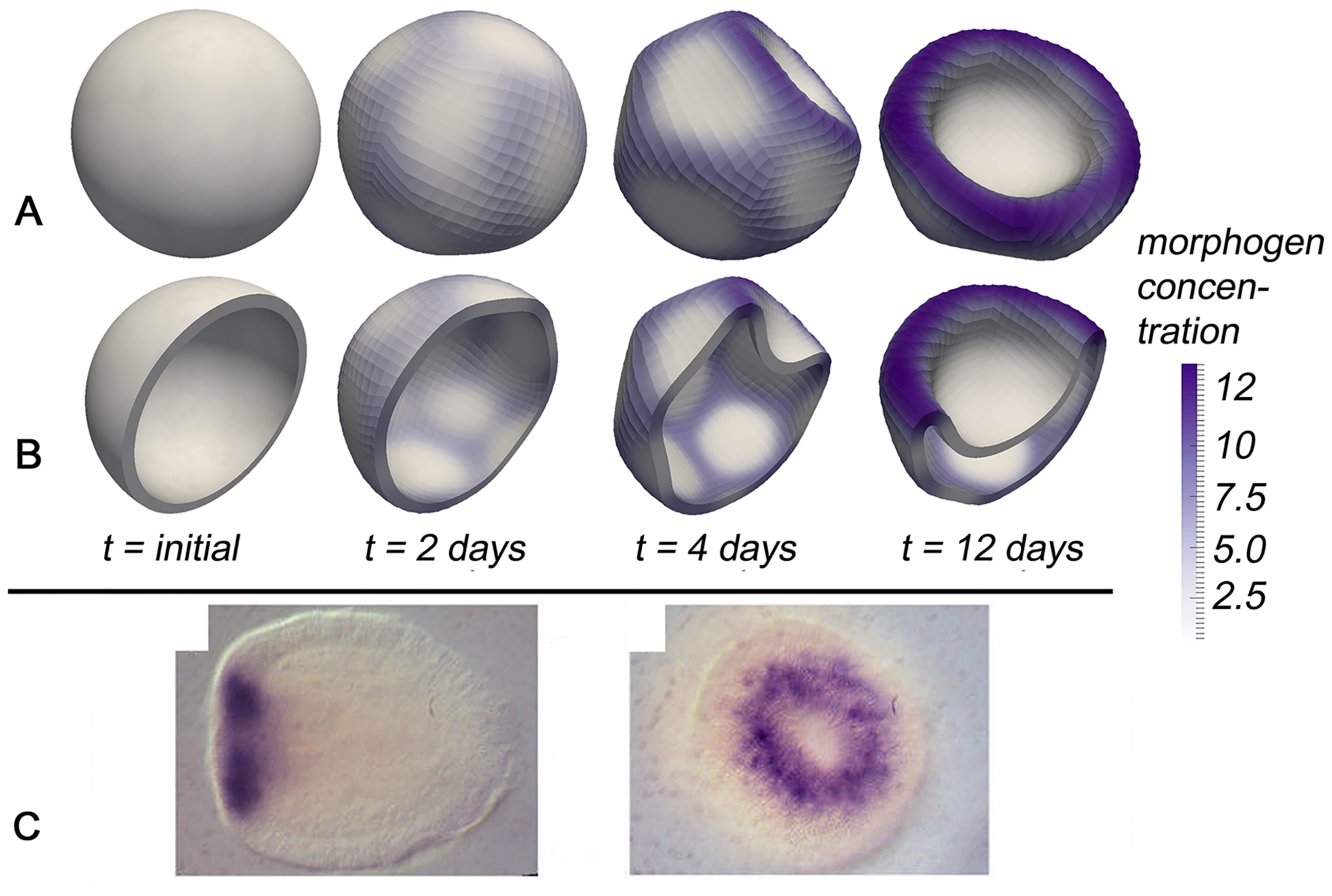
(A)-(B) Simulation snapshots showing spontaneous pattern formation based on a simple mechanochemical feedback loop including apical constriction. In (B), the 3D tissue body has been sliced just for the purpose of a better visualisation. (C) Microscopic pictures showing similar morphogen and curvature patterns in *Nematostella* during gastrulation (with permission from Ref. [76]).

Keeping all parameters constant but considering apical constriction instead of a basal one (i.e., constriction at the end of the cell pointing to the blastula lumen) finally leads to a gastrulation event, with the highest morphogen concentration found in an annulus around the invagination (cf., Fig 3A and 3B). However, due to strong deformations, the material model finally breaks down and the Newton’s method no longer converges, so that the final pattern in Fig 3A and 3B does not represent a stationary result. However, it appears that imposing an inner volume constraint may stabilise the invagination at early stages; further details are supplied in the Supporting Information.

Also here, the gastrulation appears to be insensitive to the initial conditions and thus appears to be very robust (Fig. C in S1 Text). Similar mechanochemical patterns have been observed experimentally during gastrulation, e.g. in *Xenopus* [75] and the freshwater polyp *Nematostella* [76] (Fig 3C). Interestingly, the simulations first show regular (though transient and weak) mechanochemical patterns, comparable to those from basal constriction, before gastrulation occurs (Fig 3A and 3B *t* = 2 − 4 days). The role of transient morphogen patterns during tissue development has recently further investigated e.g. by Ref. [77, 78]. If mechanochemical coupling is chosen as less intense (such as for a weaker coupling of stretch to the morphogen production), these transient patterns stabilise after *t* ≈ 4*days* without gastrulation and are strongly related to the patterns of basal constriction: in this case, deformations are essentially the same but the morphogen is no longer co-located with the inwards-directed deformation but co-located with the now active outward-directed deformation around these invaginations (see Fig. D in S1 Text for a better illustration of these similarities). In contrast, with a stronger coupling between morphogens and mechanics (as in stronger impact of stretch on the morphogen production), the relative intensity of these transient patterns diminishes.

We point out that our simulations indicate that also with basal constriction, a gastrulation (one dominating wavelength) can be obtained when mechanochemical coupling is chosen as more intense. However, numerical calculations break down at an earlier stage (c.f. Supporting Information for more information).

**Fig 4:**
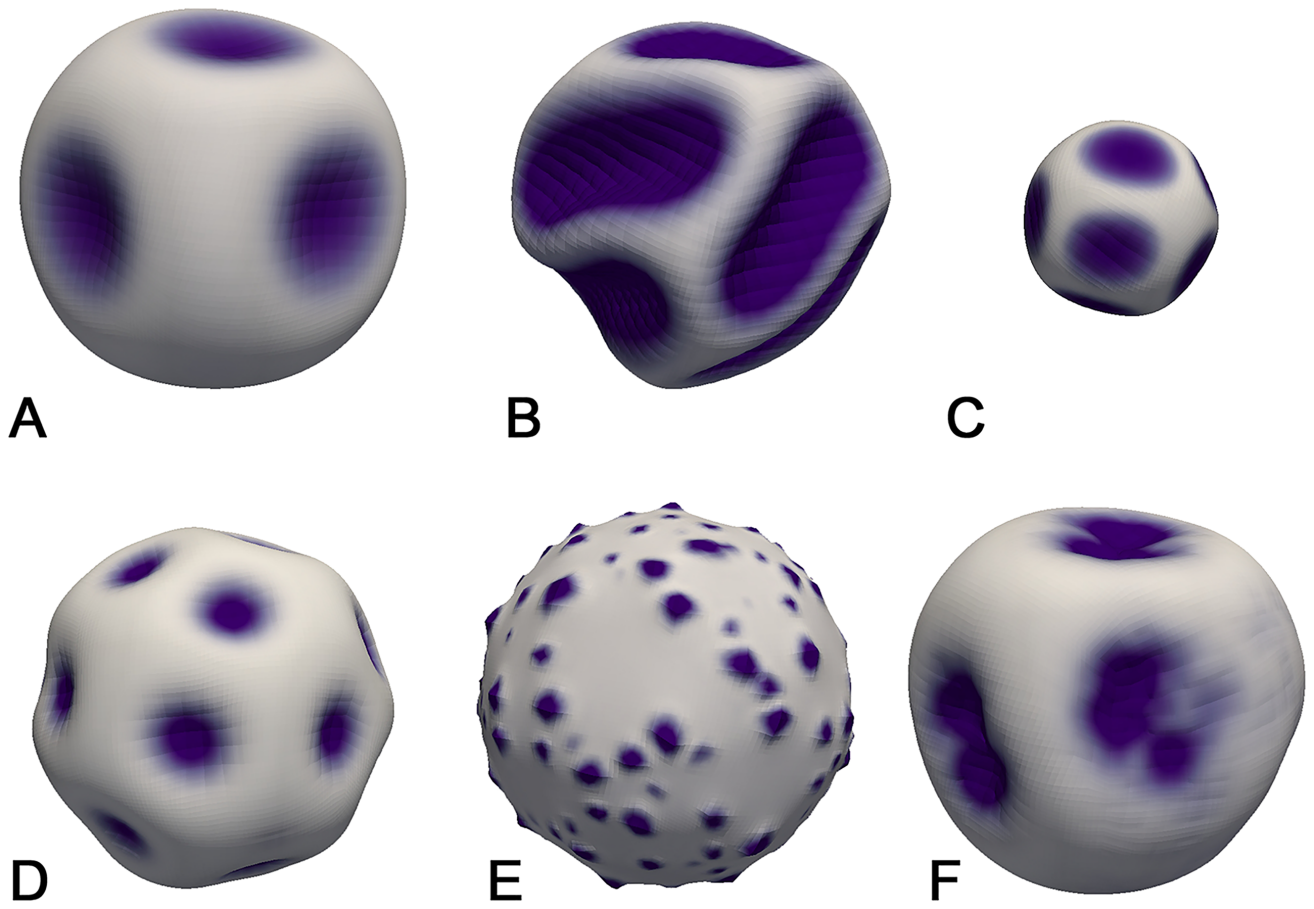
Simulation snapshots investigating the robustness of mechanochemical patterns with respect to (A) a thicker tissue layer (doubled thickness); (B) a thinner tissue layer (halved thickness); (C) a smaller system (384 biological cells); (D) lower tangential diffusion (quartered); (E) without any tangential diffusion; and (F) basal constriction only in the outer half of biological cells.

To investigate robustness of the obtained mechanochemical patterns, we performed additional simulations: First, we focused on the influence of model size and geometry on the resulting patterns. In particular, we varied the initial size of the system and the tissue thickness (relative to the radius). It appears that a thicker tissue layer leads to an increased distance between two neighbouring tissue/curvature patches whereas a thinner tissue layer leads to a decrease in the distance (Fig 4A and 4B). This observation was intuitively expected, since as explained above, mechanics is responsible for a long-range inhibition. And the stiffer the material is, the larger is a range of propagation of the mechanical signal.

Furthermore, the number of patches appears to scale with the system size: A smaller system exhibits fewer patches (Fig 4C), which is most probably a direct result of the smaller tissue surface. Also, the relative tissue thickness is increased so that patterns dominate larger parts of the domain, similarly to the situation we observed in Fig 4A.

Further, the tangential morphogen diffusion is not a critical ingredient for obtaining mechanochemical patterns: quartering the lateral diffusion strength still leads to patterns (Fig 4D). However, morphogen patches are distinctly smaller. This effect is even strengthened when tangential diffusion is completely inhibited (Fig 4E). Thus, the tangential diffusion appears to influence the size of morphogen/curvature patches.

Finally, we show that we continue to obtain mechanochemical patterns if we change the nature of the active deformations. For example if we redefine the active deformation tensor so that it constricts one side of the cell without actively expanding the other side (i.e., the active deformation is no longer volume preserving), regular patterns persist. Interestingly, morphogen patches are frequently not spherical but rather develop to “double-patches”, i.e., two superimposed patches constituting together a dumbbell-shape (Fig 4F). More details concerning various robustness tests can be found in the “Robustness of pattern formation” Section in the Supporting Information).

Our numerical simulations indicate that it is important to use a full 3D approach, since the resulting patterns presented in this work cannot be obtained by lower-dimensional approaches [3, 49]. In the three-dimensional setting of this work, we show that gastrulation-like deformations can be obtained from a range of non-uniform or non-rotationally invariant types of initial conditions, for both basal and apical constriction, and for different feedback loops based on compression or strain (Supporting Information). In 2D or pseudo 3D approaches, however, it was not possible to obtain gastrulations driven by *de novo* pattern formation [3, 49]. Hence, the full 3D approach most likely leads to more realistic simulation results, which is due to the fact that both chemical and mechanical behaviour is strongly biased if dimensions are neglected, as also explained in the Introduction. Also numerically, the difference between full 3D models and 2D approaches is significant, since the problem size increases drastically and the problem is coupled even stronger. In particular, it requires robust and parallel solvers to efficiently solve the presented 3D problems (see “Solvers and parallelisation” for details). Further, various implementation aspects changed, such as tracking biological cells via material-IDs, in particular during the domain decomposition in parallelisation.

Finally, the present study offers for the first time the possibility to explain gastrulation by robust *de novo* mechanochemical pattern formation, leading to simulation results similar to patterns observed in model organisms such as *Hydra*, *Nematostella* and *Xenopus*. However, until now, the experimental evidence for the specific feedback loops as presented in our work is still sparse. Possible reasons are: (1) visualisation of mechanical measures in living biological tissues is still under development and connected with a high experimental effort [41, 73]; and (2) the “pure chemical approach” to explain pattern formation is still very prominent among development researchers.

Encouragingly, the number of mechanochemical feedback loops experimentally documented to be drivers of pattern formation increases [50, 73, 79] and new methods of the visualisation of mechanical cues are currently under development [60–63].

Thus, our simulation results show that even simple interactions between chemistry (morphogens) and tissue mechanics can lead to robust and spontaneous pattern formation. Especially, it is worth pointing out that

- mechanochemical pattern formation appears to be very robust against perturbation of preceding patterning stages, model parameters and a range of specific assumptions;
- experimental verification of long-range inhibitors is neither necessary nor possible, since long-range inhibition may be caused by mechanical cues;
- mechanical cues (such as compression) naturally propagate at an enormous speed due to the direct mechanical interaction of molecules or cells. Hence, the mechanochemical theory is not restricted to relatively small length scale due to maximum possible diffusion rates of morphogens;
- dynamic and complex tissue topologies do not prevent patterning but are rather actively involved in pattern formation. Thus, they provide a natural and robust feedback to ensure the success of mechanical pattern formation;
- even simple linear relationships between chemistry and morphogens lead to spontaneous pattern formation, highly nonlinear interactions do not have to be assumed.

Hence, the presented mechanochemical mechanism fits well the recent experimental data and all main difficulties of purely chemical theories (cf., “Introduction”) are naturally resolved.

### Summary

In summary, the present work consist of two main segments:

1. A 3D computational approach to study mechanochemical pattern formation in developing tissues on the tissue scale: The presented method blends an explicit description of active deformations of individual, biological cells with a continuum mechanical formulation of the cell plasma. This allows us to benefit from the advantages of both approaches, e.g. combining continuous processes (such as diffusion or mechanical gradients) with the discrete nature (and possibly resulting discontinuities) of biological cells. Simulations methods are based on state of the art finite element methods (FEM) including multigrid methods, the possibility of adaptive mesh refinement, as well as parallelisation in order to minimise computational effort and to maximise numerical stability. It appears that the full 3D representation of the tissue is critical in order to obtain a realistic mechanochemical behaviour.
  During the last decade, experimental insights elucidating the role of mechanochemistry in tissue development [44–46] and experimental techniques of visualisation of mechanical loads in biological tissues [80–82] have been rapidly evolving. Hence, the presented approach may serve as a future basis of enhance interactions of experiments with simulation methods in order to further unravel one of the big mysteries in development: the self-organised generation of patterns and shapes.
2. Application of the presented framework by studying *in silico* examples of the impact of mechanochemical interactions on tissue pattern formation. Our simulations show that even simple interactions between a morphogen and tissue mechanics can lead to robust and spontaneous mechanochemical patterns, comparable to those observed during embryogenesis. Additionally, we argue that with such mechanochemical patterning mechanisms, several contradictions and difficulties arising in purely chemical patterning theories dissolve naturally. Hence, these results demonstrate the high capacity of mechanochemical interactions during tissue development—a possibly still underestimated driving force of embryonic pattern formation.

## Materials and methods

### Model geometry

**Fig 5:**
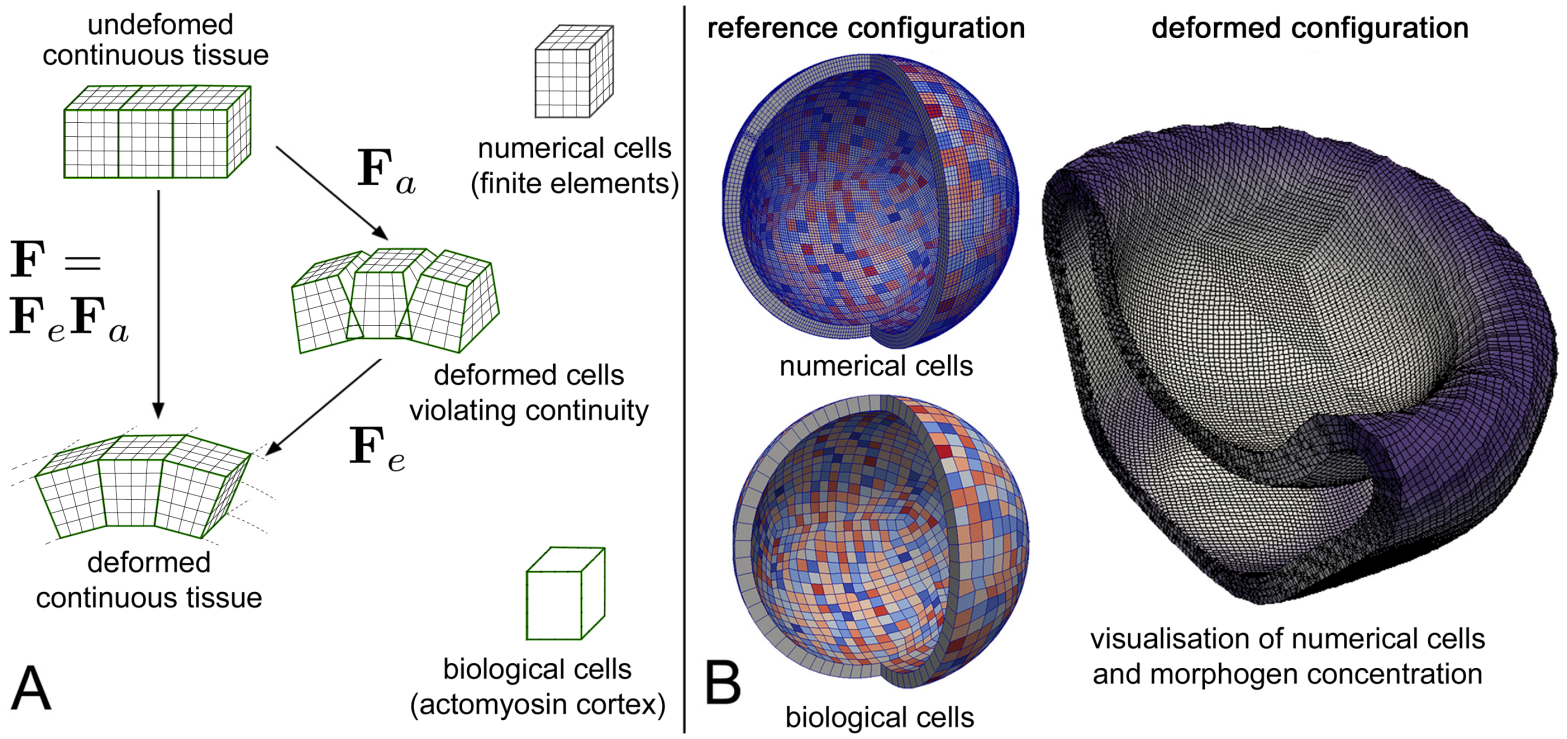
(A) Schematic view of the multiplicative decomposition of the deformation gradient tensor. (B) Numerical versus biological cells in the reference configuration (left-hand side) and an example of the deformed simulated tissue body with chemical (morphogen) patterns (right-hand side). Purple color represents high, white color low local morphogen levels. The 3D tissue body has been sliced just for the purpose of a better visualisation.

We investigate a system representing developmental stages following the blastula stage of an embryo. We parametrise a deforming tissue body over a hollow tissue sphere (cf., Fig 5B). The sphere is composed of 1536 circumferentially arranged biological cells, where each biological cell contains 64 = 4^3^ numerical cells or finite elements (cf., Fig 5). Results which stem from this discretisation have been verified using linear and quadratic finite elements using 512 = 8^3^ numerical cells per biological cell. If not stated otherwise, the outer radius of this tissue sphere is 150 *μm*, and the inner radius is 135 *μm*, resulting in tissue thickness of 15 *μm*.

### Continuum mechanics and model equations

In this subsection, we briefly introduce our model equations as well as some underlying continuum mechanical notations and relationships. For more details regarding underlying continuum mechanics we refer the reader to ref. [83]; a more detailed motivation and derivation of the model equations can be found in the Supporting Information.

#### General notation

In the following, structural dynamics are mainly expressed in the *Lagrangian* or particle-centered framework. Let **X** be a particle in the undeformed initial configuration Ω and **x** = **x**(**X**, *t*) be its current position in the deformed one Ω(*t*) at time *t*. Thus the deformation is defined by X:Ω×I→Ω(t) (with x=X(X,t)) and is assumed to be invertible as well as continuously differentiable in space and time. Furthermore, the vector fields **U**(**X**, *t*) = **x**(**X**, *t*) − **X** in Lagrangian description, or **u**(**x**, *t*) = **x** − **X**(**x**, *t*) in the Eulerian one, joining these positions is called *displacement*. These definitions directly yield **U**(**x**, *t*) = **u**(**x**, *t*). Next, **F** is the *deformation gradient* which is defined as F(X,t)≔∇X(X,t), where *J*(**X**, *t*) ≔ det(**F**(**X**, *t*)) describes the local volume change of the tissue. The *right hand Cauchy-Green tensor* is now given by **C** ≔ **F**^*T*^ **F** and the *Green-Lagrange strain tensor* by $\text{E}≔\frac{1}{2}(\text{C}-\text{I})=\frac{1}{2}({\text{F}}^{T}\text{F}-\text{I})$ Furthermore, if we denote an infinitesimal force acting on a surface element by d**f**, then the *Cauchy stress tensor* ***σ***(**x**, *t*) and the *first Piola-Kirchhoff stress tensor* **P**(**X**, *t*) are given by the relationship d*f* = ***σ***(**x**, *t*)**n**d*s* = **P**(**X**, *t*)**N**d*S*, where d*S* and d*s* depict the surface elements and **N** and **n** the normal vectors related to the reference and the current configuration, respectively. Finally, we introduce Σ(**X**) = **F**^−1^(**x**, *t*)**P**(**X**, *t*) which is the transformation of **P** to the reference configuration and is called *second Piola-Kirchhoff stress tensor*. For more notational details we refer the reader to the Supporting Information and ref. [84] and [83] (especially chapter 3).

#### Growth / Active deformations

To describe active deformation processes (such as tissue growth or active cell-shape changes) we will follow the idea of Rodriguez et al. [85, 86] and multiplicatively decompose the deformation gradient by

$$\begin{array}{c} \text{F}={\text{F}}_{e}{\text{F}}_{a} \end{array}$$ (1)

into an active part **F**_*a*_ (which maps the reference domain to an artificial intermediate configuration Ω_*a*_) and an elastic part **F**_*e*_, which ensures continuity (cf., Fig 5A). The key assumption is that the intermediate configuration is stress-free and any stress is solely generated by the elastic response **F**_*e*_. This implies, that the material law depends on the elastic response alone.

#### Mechanochemical model equations

The following equations are based on the conservation principles of mass and momentum. Additionally, we describe the tissue via a nonlinear Saint-Venant-Kirchhoff model for compressible, isotropic, hyperelastic materials. Although mechanical isotropy of blastula stages does not always occur [87], here, we assume isotropic tissue (1) for model simplicity; and (2) to prevent the introduction of pre-patterns to our model. Finally, we assume local mass conservation for diffusing and reacting morphogens in the tissue at concentration *C*. The final problem reads:

Find displacement **u** and molecule concentration *C* with initial conditions **u**(**X**, 0) = 0 and *C*(**X**, 0) = *C*^0^ such that

$$\begin{array}{ll} -\nabla \cdot (\text{F}\Sigma )=0 & \text{in}\;\Omega ,\\ J\partial_{t}C-\nabla \cdot (J{\text{F}}^{-1}\text{D}{\text{F}}^{-T}\nabla C)-JR=0 & \text{in}\;\Omega ,\\ \text{F}\Sigma \text{N}=0 & \text{on}\;\partial \Omega , \end{array}$$ (2)

holds, where

$$\begin{array}{c} \Sigma =J_{a}{\text{F}}_{a}^{-1}\Sigma_{e}{\text{F}}_{a}^{-T},\;\Sigma_{e}=\lambda \mathrm{tr}\left(E_{e}\right)I+2\mu E_{e},\;E_{e}=\frac{1}{2}\left({\text{F}}_{e}^{T}{\text{F}}_{e}-I\right),\\ {\text{F}}_{e}=\text{F}{\text{F}}_{a}^{-1},\;J=\det\left(\text{F}\right)\;\text{and}\;J_{a}=\det\left({\text{F}}_{a}\right). \end{array}$$

Here, the boundary of Ω is denoted by ∂Ω where homogeneous Neumann boundary conditions are assumed since no bounday forces are applied. Further, *μ*, λ are the Lamé constants. *R* is the coupling term incorporating the feedback of mechanical cues on the concentration *C* via *R* = *R*(**Σ**,**E**,**F**, *C*) and **Σ** = **Σ**(**F**_*a*_(*t*, *C*)) allows a reverse coupling, namely a possible influence of morphogens on active deformation processes such as local tissue growth or cell-shape changes. Finally, $D\in {\mathbb{R}}^{d\times d}$ is the diffusion coefficient matrix given by

$$\begin{array}{c} D≔R{\left(X\right)}^{T}\mathrm{diag}\left(D_{N},D_{T},D_{T}\right)R\left(X\right) \end{array}$$ (3)

with a diagonal matrix containing the diffusion coefficient *D*_**N**_ in normal (or radial), Lagrangian direction **N** = |**X**|^−1^ **X** and the diffusion coefficient *D*_**T**_ in the tangential directions **T**_1_ and **T**_2_. The rotation matrix **R**^*T*^ transforms the diagonal matrix diag(*D*_**N**_, *D*_**T**_, *D*_**T**_) defined in the point-specific coordinate systems given by orthogonal unit vectors **N**, **T**_1_ and **T**_2_ to Euclidean coordinates (Eq. (24) in the S1 Text). This choice is biologically motivated, as a large coefficient *D*_**N**_ in radial direction ensures free diffusion inside the biological cells whereas a small coefficient *D*_**T**_ limits diffusion between biological cells (at least on the tissue scale). This approach is inexact on the cellular scale, since diffusion spreads at the same speed inside a cell as across cell boundaries. However, on the one hand, the main focus of our study is patterning on tissue scale rather than cellular scale where the anisotropic diffusion serves as an approximation. On the other hand, it appears that pattern formation takes place even without any tangential diffusion (see Fig 4E) which is thus not critical for obtaining *de novo* patterns.

#### Mechanochemical interactions

So far, Eq (2) represents a general framework investigating mechanochemical processes in biological tissues. Now, we present the concrete mechanochemical feedback loops which have been used in the simulation as presented within this work.

Based on recent experimental data, in principle, various types of coupling between tissue mechanics and morphogen dynamics are imaginable: On one hand, different types of mechanical measures have been shown to influence morphogen dynamics, namely stress [41, 81, 88], compression and stretch [39, 40] and geometric constraints determining the strain and cell-shape [89, 90]. On the other hand, signalling molecules have been shown to induce a range of possible mechanical changes within tissues, such as local tissue growth, changes in tissue stiffness, or active modifications of the cell shape [73, 91–94]. However, in this work, we focus on two most frequently observed relationships between morphogens and tissue mechanics, namely morphogen inducing (basal/apical) cell constriction as well as tissue compression/stretch inducing local morphogen production.

#### Feedback of mechanics on chemistry

If we want to model an influence of a mechanical measure (such as strain, stress, or compression) on morphogen dynamics, the mechanical cues should be based on invariants *I*_*j*_, *j* ∈ {1, 2, 3} of the corresponding tensors, since these invariants do not change with the rotation of the coordinate system. As motivated within the Supporting Information in detail, the invariants of the elastic second Piola-Kirchhoff stress tensor **Σ** and of **E** as well as of **F** are suitable for a mechanical feedback on the production of signalling molecules, i.e.,

$$\begin{array}{c} R=R(I_{j}\left(\Sigma \right),I_{j}\left(E\right),I_{j}\left(\text{F}\right),C),\;j\in \left\{1,2,3\right\}. \end{array}$$

Within this work we use the determinant of the deformation gradient *I*_3_(**F**) = det(**F**) as an example of the feedback of mechanics on chemistry. This is based on experimental results [39, 68] and has the physical interpretation of compression or stretch (det(**F**) = *dv*(*t*)/*dV* is the ratio of the deformed volume element *dv*(*t*) to the initial one *dV*). However, in principle, many other modes of dependence on deformation tensor invariants are possible (some of them have been investigated in [3]).

In practice, the tensor invariant *I*_3_(**F**) is included via the Michaelis-Menten kinetics [95, 96] by

$$\begin{array}{c} R\left(I_{3}\left(\text{F}\right),C\right)=k_{2}\frac{\max\{\left(I_{3}\left(\text{F}\right)-1\right),0\}}{k_{m}+\max\left\{\left(I_{3}\left(\text{F}\right)-1\right),0\right\}}-k_{1}C, \end{array}$$ (4)

with positive constants *k*_1_, *k*_2_, *k*_*m*_ > 0. Here, *k*_1_ represents a constant degradation rate of the morphogens in the entire tissue and *k*_2_ represents its mechanically induced production. Especially, the nonlinear form of the production represents a saturation effect of *I*_3_(**F**)-induced morphogen production, which is a frequent biochemical modelling assumption due to existence of maximal production and translation rates for gene products [95, 96].

We point out that this modelling approach implies that cells “remember” their initial shape, which could be unrealistic in the view that the cell cytoskeleton is continuously remodelled (this limitation applies to all studies using finite elasticity and the multiplicative decomposition). We will consider models where the reference configuration is continuously updated in future study.

#### Coupling of chemistry with mechanics

Chemical molecules can influence local tissue mechanics in various ways, e.g. by modifying tissue stiffness, inducing local tissue growth, by actively changing the cell shape, or by combinations of these processes. Additionally, all these processes can appear in an isotropic or in an anisotropic manner. In this work, we focus on an active deformation process called apical or basal constriction, since this is a common deformation process during tissue morphogenesis [64–67]. Here, apical constriction refers to cells constricting at the side pointing *towards* the blastula lumen, whereas basal constriction concerns the cell side pointing *away* from the lumen. Mathematically, this kind of deformation is expressed by the active part of the deformation gradient tensor. Firstly, we introduce local coordinate systems $\hat{\text{X}}$ in the origin **m** of every biological cell, oriented such that ${\hat{\text{X}}}_{2}$ points in the radial direction. By **Q** and **m** we denote the rotation and the translation from the reference coordinates **X** to these parametric ones. Here, the local constriction tensor in three dimensions is given by

$$\begin{array}{c} {\hat{\text{F}}}_{a}\left(\hat{X},C\right)(\begin{array}{ccc} 1+kC{\hat{X}}_{2} & 0 & kC{\hat{X}}_{0}\\ 0 & 1+kC{\hat{X}}_{2} & kC{\hat{X}}_{1}\\ 0 & 0 & 1 \end{array}), \end{array}$$ (5)

where *k* is a constant and ${\hat{X}}_{0},{\hat{X}}_{1},{\hat{X}}_{2}$ are 3D coordinates in the cell-wise reference system. For positive values of *k*, this results in apical constriction and for negative values in a basal one. Observing **QQ**^*T*^ = **Q**^*T*^ **Q** = **I**, **F**_*a*_ in the reference system is now given as the transformation of the tensor and its argument by

$$\begin{array}{c} {\text{F}}_{a}\left(X,C\right)=Q^{T}{\hat{\text{F}}}_{a}\left(QX-Qm,C\right)Q. \end{array}$$ (6)

Note that **Q** depends on the biological cell under consideration whereas ${\hat{\text{F}}}_{a}$ remains identical. We have depicted the local coordinate systems $\hat{\text{X}}$ for a biological cell *K*_*i*_ and the transformation **Q** in the Supporting Information (cf. Fig. A in S1 Text). In particular, this means that **F**_*a*_ is a piecewise-defined tensor which results in a semi-discrete model whereas, Eq (2) was entirely continuous up to this point. This specific choice of ${\hat{\text{F}}}_{a}$ and thus **F**_*a*_ (since det(**Q**) = 1) is volume-preserving since for the deformed volume ${\hat{V}}_{i,a}$ (deformed by ${\hat{\text{F}}}_{a}$) and initial volume ${\hat{V}}_{i}$ of any biological cells *K*_*i*_ it holds

$$\begin{array}{c} \begin{array}{cc} {\hat{V}}_{i,a} & =\int_{K_{i,a}}d{\hat{X}}_{2}d{\hat{X}}_{1}d{\hat{X}}_{0}=\int_{K_{i}}\left|\det\left({\hat{\text{F}}}_{a}\right)\right|d{\hat{X}}_{2}d{\hat{X}}_{1}d{\hat{X}}_{0}\\ & =\int_{K_{i}}{\left(1+kC{\hat{X}}_{2}\right)}^{2}d{\hat{X}}_{2}d{\hat{X}}_{1}d{\hat{X}}_{0}={\hat{V}}_{i}+kC\int_{K_{i}}{\hat{X}}_{2}\left(2+kc{\hat{X}}_{2}\right)d{\hat{X}}_{2}d{\hat{X}}_{1}d{\hat{X}}_{0}={\hat{V}}_{i}, \end{array} \end{array}$$

see Fig. A in S1 Text. The last integral vanishes since the centroid of *K*_*i*_ has been transformed to the origin and integration with respect to ${\hat{X}}_{1}$ and ${\hat{X}}_{0}$ cancels out.

Active constriction processes usually occur on one side of a biological cell by local contraction of correspondingly located acto-myosin networks [65]. Nevertheless, during these deformations, the local volume of the cells often appears to be conserved [97] so that we choose **F**_*a*_ volume preserving as described above and similarly as in Conte *et al.* [98]. The robustness of patterns with respect to alternatively defined active deformations (non-volume preserving or continuously defined *F*_*a*_) can be found in Fig 4F respectively in the S1 Text.

Finally, we point out that although cell boundaries have been considered explicitly (in order to appropriately describe active deformations such as constriction), the presented model is designed to be realistic at the tissue scale rather than the cell scale, since there is no resolution of sub-cellular structures.

#### Mechanochemical feedback loop

Mechanochemical patterns are formed by a positive feedback loop as sketched in Fig 1. This feedback loop is based on the two experimentally motivated assumptions:

1. local morphogen levels lead to apical constriction of individual, biological cells [65] (which again leads to local stretch/compression of the tissue due its elastic response), and finally
2. local stretch and compression induce local morphogen production [68, 81].

By this construction, this feedback loop is self-energising and the Michaelis-Menten kinetics Eq (4) ensure that the process slows down and eventually becomes stationary. Results regarding alternative feedback loops are given in the Supporting Information.

### Finite element (FEM) approximation

#### Discretisation

For the finite element approximation, our domain Ω is split into 98304 numerical cells. This is due to the initial 3D-sphere Ω_*H*_ which is composed of 1536 circumferentially arranged biological cells of size *H* that are each resolved by 64 numerical cells (hexaedra) of size *h* < *H*. These cells are then pulled onto the three-dimensional sphere, such that Ω_*h*_ ≈ Ω. This discretisation in hexaedra yields 122890 nodal points and the same number of isoparametric, linear finite elements (Q1-FE) used for the approximation in space. Selected results, e.g. in Fig 3, were additionally verified on 786432 numerical cells (i.e. every biological one is split into 512 numerical ones) with a discretisation by isoparametric quadratic finite elements (Q2-FE).

Since the discrete solution **u** is continuous, the same holds true for **F** and the feedback term *R*(*I*_3_(**F**)). Thus, we do not require local mesh refinement to resolve any discontinuities in this work. In contrast, feedback terms depending on the discontinuous second Piola-Kirchhoff stress tensor **Σ** require mesh adaptivity [3].

The time derivatives are discretised by using *θ*-time-stepping methods [84] (chapter 4.1). Namely, we use the implicit Euler method for the first derivative in the reaction-diffusion equation modelling morphogen dynamics and a two-step method for the second derivative in the stabilisation term of the structural one.

In practice, the initial conditions **u**(**X**, 0) = 0, *C*(**X**, 0) = *C*^0^ are incompatible, since the initial displacement **u**(**X**, 0) corresponding to the prescribed initial concentrations *C*^0^ is usually unknown and set to zero. Hence, it takes a few time steps for deformation and concentrations to match. These first 20 to 50 time steps are computed using a smaller time step size. For this, a simple, adaptive time discretisation is employed which registers the convergence speed and automatically increases the size of the time steps if deformation and concentrations begin to match. That way, fast convergence of Newton’s method is always ensured.

#### Solvers and parallelisation

In three dimensions, the system matrix to solve in each time step is strongly coupled and of considerable size. Efficiently solving our equations in three dimensions requires parallelised, state of the art solution techniques. In the first step, the system (2) is linearised by Newton’s method. The resulting, linear system in each of the 2-3 Newton steps, depending on the current convergence rate, is then solved by 8-10 GMRES iterations [99]. The latter method is preconditioned by a geometric multigrid method [100]. The main computational effort of the multigrid method is the smoother which reduces the high frequent error components on the corresponding grid. Thus, a parallelised ILU factorisation was used as smoother in all computations. The parallelisation was done using MPI and is based on the idea of decomposing the domain Ω_*h*_ in *n* parts of similar size and with minimal overlap. The ILU decomposition is applied on each part individually and the solution is subsequently combined, for details we refer to Ref. [101, 102]. For *n* parts, we thus use *n* CPU cores. On an Intel(R) Xeon(R) CPU E5-2690 0 @ 2.90 GHz with 16 physical cores (32 by hyperthreading) and 256 GB RAM, the computation time for one time step reduces from 49 seconds per time step averaged over 1000 steps to 24 seconds on two cores and only 4 seconds on 14 cores. Note that the efficiency of the parallelisation decreases the more cores are used. The largest gain occurs by using two instead of one core. Still, an impressive reduction of computational costs by a factor 12 is possible by solving on 14 cores (two of the 16 were used for the operating system as well as the process that steers the parallelisation).

The bottleneck is the mere number of time steps required to resolve the short elastic timescale introduced by a time derivative in the structural equation which is added for stabilisation purposes. Details are given in the Supporting Information. For the numerical results in Fig 2 and in Fig 3, 100000 and 50000 time steps were required respectively. On the server mentioned previously, this requires about 4 and accordingly 2 days to obtain these results if all parameters are known and the machine is otherwise unoccupied.

The convergence of our method was verified on a finer mesh for linear and quadratic finite elements as stated above. Thereby, it was shown, that the stabilisation error is of smaller scale than the discretisation error, i.e. the stabilisation does not effect the quality of the results.

### Parameter setup

We begin with the essential parameters: Mathematically and biologically, it is vital to have a comparably small diffusion coefficient *D*_**T**_ ∼ 10^−14^ *m*^2^ *s*^−1^ in the tangential directions in relation to a large coefficient *D*_**N**_ ∼ 10^−12^ *m*^2^ *s*^−1^ in the normal one. Now, the crucial point in finding suitable parameters is to balance the choice of the diffusion coefficients with the maximal morphogen production rate *k*_2_ ∼ 10^7^ *molm*^−3^ *s*^−1^ which is also related to the Michaelis constant which was set to *k*_*m*_ = 2.0. Once the stretch has reached this value, i.e. *I*_*e*_(**F**) = 2.0, half of the production rate *k*_2_ is reached. The latter two parameters *k*_2_ and *k*_*m*_ depend on one another as they both influence maximal morphogen concentration after saturation in conjunction with the diffusion coefficients. Further, we set *k*_1_ ∼ 10^−4^ *s*^−1^ for the degradation rate of the morphogen level in the entire domain. Detailed numerical studies how changes in these parameters affect resulting patterns can be found in the Supporting Information (“Robustness of pattern formation”).

Notably, the choice of the material constants is not essential. This can be seen as follows: Values of the Lamé constants are usually given in terms of Young’s modulus *E* and Poisson’s ratio *ν*. They can be obtained by the conversion formulas given in Eq. (18) in the S1 Text. In particular, the Lamé constants linearly depend on *E*. A dimensionless analysis shows that, in the absence of external forces, Young’s modulus can be extracted from the structural equation and changes in *E* only alter the elastic timescale, which is not resolved since we are only interested in the comparably long timescale of active deformations (cf. section “Full mechanochemical model equations” in the Supporting Information for details on the time scale.) Also, changing *ν* does not significantly alter the results. We confirmed this numerically since we have we used *E* = 100 *Pa* and *ν* = 0.4 as in [103] for our computations but choosing *E* = 1000 *Pa* and *ν* = 0.3 as in [98] leads to qualitatively the same results.

Finally, uniformly distributed random concentrations for each biological cell or a morphogen gradient were used as initial conditions. In both cases, the morphogen concentration was included in the interval *c* ∈ [0, 10^9^]mol m^−3^. For visualisation of the following results, initial conditions were transformed into the interval *c* ∈ [0, 1]mol m^−3^. In any case, the scale of the morphogen concentration is not crucial since only the constant *k* which determines how strong the morphogen concentration couples into the active deformation gradient (see Eq (5)), has to scale in the same manner. It was set to *k* ∼ 10^−6^ *mol*^−1^ *m*.

## Supporting information

S1 Text PDF document comprising additional information (text and figures) with respect to structural mechanics and notation, structural model equations, implementation of growth, full mechanochemical model equations, derivation of mechanical invariants as well as robustness of pattern formation. (PDF)
