# Supplementary material for: Post-Turing tissue pattern formation: Advent of mechanochemistry
Source: PLoS Comput Biol. 2018 Jul 3;14(7):e1006259. doi: 10.1371/journal.pcbi.1006259 (PMC6047832; doi:10.1371/journal.pcbi.1006259)
Supplement: S1 Text — (PDF) [file pcbi.1006259.s001.pdf]

# SUPPORTING INFORMATION S1

## Post-Turing tissue pattern formation: Advent of Mechanochemistry

Felix Brinkmann[1,+], Moritz Mercker[1,+,*], Thomas Richter[2],Anna Marciniak-Czochra[1]
**1 Institute of Applied Mathematics, BioQuant and Interdisciplinary Center of Scientific Computing (IWR), Heidelberg University, Heidelberg, Germany**
**2 Magdeburg University, Institute for Analysis and Numerics, Magdeburg, Germany**
+ ***Both authors contribute equally to this work***
∗ **E-mail: mmercker_bioscience@gmx.de**

# Structural mechanics and notation

In the following, structural dynamics are mainly expressed in the *Lagrangian* or particle-centered framework. Let $\mathbf{X}$ be a particle in the undeformed initial configuration $\Omega$ and $\mathbf{x} = \mathbf{x}(\mathbf{X}, t)$ be its current position in the deformed one $\Omega(t)$ at time $t$. Thus the deformation is defined by $\boldsymbol{\chi}\colon \Omega \times I \to \Omega(t)$ (with $\mathbf{x} = \boldsymbol{\chi}(\mathbf{X}, t)$) and is assumed to be invertible as well as continuously differentiable in space and time. Furthermore, the vector field

$$\mathbf{U}(\mathbf{X}, t) = \mathbf{x}(\mathbf{X}, t) - \mathbf{X}$$

in Lagrangian description, or

$$\mathbf{u}(\mathbf{x}, t) = \mathbf{x} - \mathbf{X}(\mathbf{x}, t)$$

in the Eulerian one, joining these positions is called *displacement.* This directly yields the relation

$$\mathbf{u}(\mathbf{x}, t) = \mathbf{U}(\boldsymbol{\chi}^{-1}(\mathbf{x}, t), t) = \mathbf{U}(\mathbf{X}, t) \tag{7}$$

which allows us to state all results in terms of $\mathbf{u}$ in the following. The same holds for the velocity field $\mathbf{V}(\mathbf{X}, t)$. Denoting the *material line element* to a material curve by $d\mathbf{X}$ and a *spatial line element* or spatial tangent vector to a spatial curve by $d\mathbf{x}$, then we obtain the important relation for the local change of relative positions by

$$d\mathbf{x} = \mathbf{F}(\mathbf{X}, t) d\mathbf{X},$$

where $\mathbf{F}$ is the *deformation gradient* which is defined as

$$\mathbf{F}(\mathbf{X}, t) := \nabla\boldsymbol{\chi}(\mathbf{X}, t) = \nabla\mathbf{u}(\mathbf{x}, t) + \mathbf{I}, \quad J(\mathbf{X}, t) := \det(\mathbf{F}(\mathbf{X}, t)),$$

with the identity matrix $\mathbf{I}$. $J$ represents the relative change of the local tissue volume and $\mathbf{F}$ plays an important role when we consider active deformations such as growth (c.f., following section). Based on $\mathbf{F}$, the following strain tensors can be derived: *The right hand Cauchy-Green tensor*

$$\mathbf{C} := \mathbf{F}^T\mathbf{F}, \text{ with } \det(\mathbf{C}) = \det(\mathbf{F})^2 = J^2, \tag{8}$$

as well as the *Green-Lagrange strain tensor*

$$\mathbf{E} := \frac{1}{2}(\mathbf{C} - \mathbf{I}) = \frac{1}{2}(\mathbf{F}^T\mathbf{F} - \mathbf{I}), \tag{9}$$

both representing a measure of change of volume respectively shape. For further details on strain tensors in the material configuration we refer to Holzapfel [1], section 2.5.

Furthermore, we denote an infinitesimal force acting on a surface element by $\mathrm{d}\mathbf{f}$ and surface elements by $\mathrm{d}S$ and $\mathrm{d}s$ and normal directions by $\mathbf{N}$ and $\mathbf{n}$ in the reference and the current configuration, respectively. Then, we claim that there exist unique second order tensor fields $\boldsymbol{\sigma}(\mathbf{x}, t)$, $\mathbf{P}(\mathbf{X}, t)$ (Cauchy's stress theorem) such that

$$\mathrm{d}\mathbf{f} = \boldsymbol{\sigma}(\mathbf{x}, t)\mathbf{n}\mathrm{d}s = \mathbf{P}(\mathbf{X}, t)\mathbf{N}\mathrm{d}S, \tag{10}$$

where $\boldsymbol{\sigma}(\mathbf{x}, t)$ is the *Cauchy stress tensor* and $\mathbf{P}(\mathbf{X}, t)$ the *first Piola-Kirchhoff stress tensor*. Finally, we introduce

$$\boldsymbol{\Sigma}(\mathbf{X}) = \mathbf{F}^{-1}(\mathbf{x}, t)\mathbf{P}(\mathbf{X}, t) \tag{11}$$

which is the transformation of $\mathbf{P}$ to the reference configuration and is called *second Piola-Kirchhoff stress tensor* and expresses the stress solely in the reference configuration. All three stress tensors are related via

$$\mathbf{P} = J\boldsymbol{\sigma}\mathbf{F}^{-T} = \mathbf{F}\boldsymbol{\Sigma}; \tag{12}$$

for more details on the concept of stress we refer to textbooks, e.g. Holzapfel [1], chapter 3.

# Structural model equations

The shape of structural equations is determined by the two principles of conservation of mass and momentum. These balance principles are naturally given in the current configuration $\Omega(t)$. In strong formulation, they read

$$\rho = J\rho^0, \qquad \text{in } \Omega(t), \tag{13}$$

$$\rho\partial_{tt}\mathbf{u} = \rho\mathbf{f} + \nabla\cdot\boldsymbol{\sigma} \quad \text{in } \Omega(t), \tag{14}$$

for current and initial mass distributions $\rho(\mathbf{x}, t)$ and $\rho^0(\mathbf{X})$, external forces $\mathbf{f}(\mathbf{x}, t)$ and surface forces $\boldsymbol{\sigma}(\mathbf{x}, t)\mathbf{n}$ which can be expressed with the stress tensor $\boldsymbol{\sigma}$ as discussed before (Eq. (10)). The divergence comes up by the application of the Gauss's theorem to Cauchy's first equation of motion. As mentioned before, it is usually more convenient to express continuum mechanics for elastic solids in the reference configuration. Then, *Cauchy's first equation of motion in Lagrangian coordinates* holds pointwise in the reference configuration $\Omega$ and reads

$$\rho^0\partial_{tt}\mathbf{u} - \nabla\cdot(\mathbf{F}\boldsymbol{\Sigma}) = \rho^0\mathbf{f} \quad \text{in } \Omega. \tag{15}$$

We want to emphasise that this equation was derived from the physical principles of conservation of mass and momentum only. Especially, they are valid for all materials at all times and involve no additional modelling assumption. If we want to predict the material response of particular materials such as biological tissue, we additionally need to specify a material law e.g. relating the stress to the deformation gradient $\mathbf{F}$. In particular, we restrict ourselves to *hyperelastic materials* where we assume the existence of a scalar-valued stored-energy function $\Psi = \Psi(\mathbf{F})$, see e.g. Ciarlet [2] or Holzapfel [1]. There are many equivalent forms of the stored-energy function, especially it can be expressed via $\Psi(\mathbf{F}) = \Psi(\mathbf{E}) = \Psi(\mathbf{C})$. This implies, that the material is homogeneous, since $\Psi$ depends on the deformation gradient alone and not on the current position $\mathbf{X}$. Furthermore, it is assumed that our biological tissue is isotropic and isothermal. This means, that the material response is the same in all directions and for all temperatures. We obtain relations of the form

$$\mathbf{P} = \frac{\partial\Psi(\mathbf{F})}{\partial\mathbf{F}}, \quad \boldsymbol{\Sigma} = \frac{\partial\Psi(\mathbf{E})}{\partial\mathbf{E}}. \tag{16}$$

Obviously, assuming isotropy is a strong idealization: Each biological cell has a complex network of proteins called cytoskeleton, which gives a cell its shape and mechanical resistence to deformation. On the other hand, assuming isothermal behaviour is a reasonable assumption since temperature does not change significantly during biological processes such as pattern formation.

In the following, we use the simple nonlinear Saint-Venant-Kirchhoff model for compressible, hyperelastic materials to model the biological tissue. It states a linear relation between stress and strain, called Hooke's law. The strain energy function for the Saint-Venant-Kirchhoff model is given by

$$\Psi(\mathbf{E}) = \frac{\lambda}{2}\,\mathrm{tr}(\mathbf{E})^2\mathbf{I} + \mu\,\mathrm{tr}(\mathbf{E}^2),$$

which provides a relation between the second Piola-Kirchhoff stress tensor $\mathbf{\Sigma}$ and the Green-Lagrangian strain $\mathbf{E}$ tensor, namely

$$\mathbf{\Sigma} = \frac{\partial \Psi(\mathbf{E})}{\partial \mathbf{E}} = \lambda\,\mathrm{tr}(\mathbf{E})\mathbf{I} + 2\mu\mathbf{E}, \tag{17}$$

where $\mu$ and $\lambda$ are the Lamé constants. Values of the Lamé constants are usually given in terms of the Young's modulus $E$ which is a measure of the stiffness (relation of stress to strain along an axis) of a solid material and Poisson's ratio $\nu$ which is the signed ratio of transverse to axial strain. Both measures can be obtained by the conversion formulas

$$\mu = \frac{E}{2(1+\nu)} \text{ and } \lambda = \frac{E\nu}{(1+\nu)(1-2\nu)}. \tag{18}$$

Note, that the Poisson's ratio for typical isotropic, compressible materials has a ratio of $0.2 < \nu < 0.5$. The upper limit case $\nu = 0.5$ holds for isotropic, incompressible materials.

Now, we can combine the material law (17) and the Green-Lagrange strain tensor $\mathbf{E}$ defined in Eq. (9) with the balance principles from above, leading to the following problem: Find displacement $\mathbf{u}$ such that

$$\begin{aligned} \rho^0 \partial_{tt}\mathbf{u} - \nabla\cdot(\mathbf{F\Sigma}) &= 0 \qquad \text{in } \Omega, \\ \mathbf{F\Sigma N} &= 0 \qquad \text{on } \partial\Omega, \end{aligned} \tag{19}$$

holds, where

$$\mathbf{\Sigma} = \lambda\,\mathrm{tr}(\mathbf{E})\mathbf{I} + 2\mu\mathbf{E}, \quad \mathbf{E} = \frac{1}{2}(\mathbf{F}^T\mathbf{F} - \mathbf{I}).$$

Here, the boundary of our domain $\Omega$ is denoted by $\partial\Omega$ where homogeneous Neumann boundary conditions were assumed since no surface forces are acting on the boundary. Note that homogeneous Dirichlet values cannot be assumed anywhere on the boundary since it is a priori not known where the deformation will take place.

The latter is a considerable handicap, since the solution $u$ is no longer unique, if we intend to omit the time derivative in the previous equation. Consequently, the time derivative from above has to remain as a stabilization which will require us to perform considerably more timesteps in practice.

# Implementation of growth

As before, we use the deformation gradient decomposition approach (c.f., Eq. (1) main manuscript) to implement growth. Furthermore, we assume that the active deformation tensor $\mathbf{F}_a$ may depend on local morphogen concentrations $C$, thus $\mathbf{F}_a = \mathbf{F}_a(C)$, see also Eq. (6) main manuscript.

The key assumption behind the deformation gradient decomposition is that the intermediate state $\Omega_a$ is stress free. Thus, any stress in the material body is generated by the elastic deformation $\mathbf{F}_e$. As

a consequence, the elastic energy denisty $\Psi$ and thus the first and second Piola-Kirchhoff stress tensors depend only on $\mathbf{F}_e$:

$$\begin{aligned} \mathbf{P}_e &= \frac{d\Psi}{d\mathbf{F}_e} : \Omega_a \to \Omega(t), \\ \mathbf{\Sigma}_e &= \frac{d\Psi}{d\mathbf{E}_e} : \Omega_a \to \Omega_a, \end{aligned} \tag{20}$$

and thus

$$\mathbf{\Sigma}_e = \lambda \operatorname{tr}(\mathbf{E}_e)\mathbf{I} + 2\mu \mathbf{E}_e, \quad \mathbf{E}_e = \frac{1}{2}\left(\mathbf{F}_e^T \mathbf{F}_e - \mathbf{I}\right), \tag{21}$$

in contrast to the usual relation (16) and the material law (17).

As a consequence, $\mathbf{P}_e \mathbf{n}_a$ depicts the force in the current configuration $\Omega(t)$ given per unit surface area related to $\Omega_a$. However, the intermediate configuration is not a real domain, it might not even be continuous (e.g. if $\mathbf{F}_a$ is only piecewise continuous) and is never physically attained. Furthermore, a surface area element in $\Omega_a$ might not be uniquely defined.

Similarly, $\mathbf{\Sigma}_e \mathbf{n}_a$ depicts the force in the intermediate configuration $\Omega_a$ given per unit surface area in $\Omega_a$, which directly yields analogously to (16):

$$\mathbf{P}_e = \mathbf{F}_e \mathbf{\Sigma}_e.$$

Since $\mathbf{\Sigma}_e$ is defined on the intermediate configuration and conservation of momentum cannot be written here, we intend to transform the balance of linear momentum equation to the reference domain $\Omega$.

Transformation to the reference configuration yields the following relation between the second Piola-Kirchhoff stress tensor $\mathbf{\Sigma}$ and its elastic equivalent:

$$\mathbf{\Sigma} = J_a \mathbf{F}_a^{-1} \mathbf{\Sigma}_e \mathbf{F}_a^{-T}. \tag{22}$$

Note that both arguments of the tensor $\mathbf{\Sigma}_e$ have to be transformed from the intermediate configuration $\Omega_a$ to the reference one. Thus, the Jacobian $\mathbf{F}_a^{-1}$ of the transformation occurs twice. For a detailed derivation of this relation using tensor algebra we refer to the literature, for instance, see section 10 in Lubarda and Hoger [3].

We point out that the elastic Green-Lagrangian strain tensor $\mathbf{E}_e$ and subsequently the Cauchy stress tensor $\sigma_e$ and the second Piola-Kirchhoff tensor remain symmetric, since

$$\mathbf{E}_e^T = \frac{1}{2}\left(\mathbf{F}_a^{-T}\mathbf{F}^T\mathbf{F}\mathbf{F}_a^{-1} - \mathbf{I}\right)^T = \frac{1}{2}\left((\mathbf{F}_a^{-T}\mathbf{F}^T\mathbf{F}\mathbf{F}_a^{-1}) - \mathbf{I}\right) = \mathbf{E}_e$$

holds.

Using the relation (22) we just derived, we can finally insert $\mathbf{\Sigma}_e$ into our structural equation (19) which is given in the reference configuration. To derive our full mechanochemical system of model equations, we will now focus on the description of morphogen dynamics.

## Full mechanochemical model equations

In Eulerian coordinates, the prototypic reaction diffusion equation for a concentration of signalling molecules $c(\mathbf{x}, t)$ reads with the velocity $\mathbf{v}(\mathbf{x}, t)$:

$$\partial_t c(\mathbf{x}, t) + (\mathbf{v}(\mathbf{x}, t) \cdot \nabla) c(\mathbf{x}, t) - \nabla \cdot (\mathbf{D} \nabla c(\mathbf{x}, t)) - R(\mathbf{x}, t) = 0 \quad \text{in } \Omega(t), \tag{23}$$

with the diffusion coefficient matrix $\mathbf{D} \in \mathbb{R}^{3\times 3}$, which we will further specify. The reaction term $R(\mathbf{x}, t)$ contains the mechanical feedback on the dynamics of signalling molecules and was presented in Eq. (4) (main manuscript). We transform Eq. (23) to the reference domain $\Omega$ using the deformation $\boldsymbol{\chi} \colon \Omega \to \Omega(t)$ and the transformations $\nabla_{\mathbf{X}} C(\mathbf{X}, t) = \mathbf{F}(\mathbf{X}, t)^T \nabla_{\mathbf{x}} c(\mathbf{x}, t)$ and $\partial_t C(\mathbf{X}, t) = \partial_t c(\mathbf{x}, t) + (\mathbf{v}(\mathbf{x}, t) \cdot \nabla_{\mathbf{x}}) c(\mathbf{x}, t)$ of the gradient and the partial time derivative of a scalar-valued function $C$, respectively. The proof of these transformations is based on the chain rule and is given in standard textbooks, e.g. Richter [4]. Further, we employ the notation $C = c(\boldsymbol{\chi}(\mathbf{X}, t), t)$, $\mathbf{v} = \mathbf{v}(\boldsymbol{\chi}(\mathbf{X}, t), t)$ for the sake of a simple presentation.

The reaction-diffusion equation is now given in the reference configuration i.e. in the same framework as the structural equation (19) and reads

$$J\partial_t C - \nabla \cdot (J\mathbf{F}^{-1}\mathbf{D}\mathbf{F}^{-T}\nabla C) - JR = 0 \quad \text{in } \Omega. \tag{24}$$

As motivated in the main manuscript, we prescribe individual diffusion rates $D_{\mathbf{N}}$ in normal (or radial), Lagrangian direction $\mathbf{N} = |\mathbf{X}|^{-1}\mathbf{X}$ of the tissue sphere and $D_{\mathbf{T}}$ in tangential, Lagrangian directions $\mathbf{T}_1$ and $\mathbf{T}_2$ (c.f. Eq. (3) of the main manuscript). This yields the diffusion coefficient matrix

$$\mathbf{D} = \mathbf{R}^T \operatorname{diag}(D_{\mathbf{N}}, D_{\mathbf{T}}, D_{\mathbf{T}})\mathbf{R},$$

where the diagonal matrix $\operatorname{diag}(D_{\mathbf{N}}, D_{\mathbf{T}}, D_{\mathbf{T}})$ is expressed in the point-specific coordinate systems given by the unit vectors $\{\mathbf{N}(\mathbf{X}), \mathbf{T}_1(\mathbf{X}), \mathbf{T}_2(\mathbf{X})\}$. The matrix $\mathbf{R}(\mathbf{X})$ is the transformation of the Cartesian coordinates to these systems and can be interpreted as a rotation. The matrices are given by

$$\operatorname{diag}(D_{\mathbf{n}}, D_{\boldsymbol{\tau}}, D_{\boldsymbol{\tau}}) = \begin{pmatrix} D_{\mathbf{n}} & 0 & 0 \\ 0 & D_{\boldsymbol{\tau}} & 0 \\ 0 & 0 & D_{\boldsymbol{\tau}} \end{pmatrix} \quad \text{and} \quad \mathbf{R}(\mathbf{x}) = \begin{pmatrix} N_1 & N_2 & N_3 \\ T_{1,1} & T_{1,2} & T_{1,3} \\ T_{2,1} & T_{2,2} & T_{2,3} \end{pmatrix}.$$

However, before we combine both equations, we want to point out that we have so far neglected an important consequence of the deformation gradient decomposition as introduced above. Namely, it implies a separation of the time scale of growth / active deformations from the elastic one. Indeed, in biological tissues, growth takes place over a timescale of several hours or days whereas the elastic material response occurs within seconds. Obviously, we are interested in long-term active deformations (growth) rather than unrealistic, high frequent oscillations on the elastic time scale, which are also numerically costly to resolve i.e. it requires signigicantly smaller and thus considerably more timesteps. Consequently, we would like to neglect the time derivative $\rho^0 \partial_{tt}\mathbf{u}$ in the structural equation ("quasi-stationary formulation"). However, this leads to convergence problems of Newton's method, since the resulting problem is no longer unique as described above. Thus, we introduce the time derivative as a stabilization term with a stabilization parameter $\epsilon \sim 0.1$ to balance both effects.

Hence, the final coupled mechanochemical problem reads: Let $\Omega \subset \mathbb{R}^3$, be a (boundend) domain in two or three dimensions. Further, let the boundary of our domain be denoted by $\partial\Omega$ where homogeneous Neumann boundary conditions will be assumed. Then, find displacement $\mathbf{u}$ and morphogen concentration $C$, with initial conditions $\mathbf{u}(\mathbf{X}, 0) = 0$, $C(\mathbf{X}, 0) = C^0$ such that

$$\begin{aligned} \epsilon\rho^0\partial_{tt}\mathbf{u} - \nabla \cdot (\mathbf{F}\boldsymbol{\Sigma}) &= 0 \qquad \text{in } \Omega, \\ J\partial_t C - \nabla \cdot (J\mathbf{F}^{-1}\mathbf{D}\mathbf{F}^{-T}\nabla C) - JR &= 0 \qquad \text{in } \Omega, \\ \mathbf{F}\boldsymbol{\Sigma}\mathbf{N} &= 0 \qquad \text{on } \partial\Omega, \end{aligned} \tag{25}$$

holds, where

$$\mathbf{\Sigma} = J_a \mathbf{F}_a^{-1} \mathbf{\Sigma}_e \mathbf{F}_a^{-T}, \quad \mathbf{\Sigma}_e = \lambda \operatorname{tr}(\mathbf{E}_e)\mathbf{I} + 2\mu \mathbf{E}_e, \quad \mathbf{E}_e = \frac{1}{2}(\mathbf{F}_e^T \mathbf{F}_e - \mathbf{I}),$$
$$\mathbf{F}_e = \mathbf{F}\mathbf{F}_a^{-1}, \quad J = \det(\mathbf{F}) \text{ and } J_a = \det(\mathbf{F}_a).$$

Again, $\mu$ and $\lambda$ are the Lamé constants and $\mathbf{D} \in \mathbb{R}^{3\times 3}$ is the diffusion coefficient matrix which allows for individual diffusion rates in radial and tangential directions as derived above. The reaction term $R = R(\mathbf{\Sigma}, \mathbf{E}, \mathbf{F}, C)$ contains the mechanical feedback on the dynamics of signalling molecules and was presented in Eq. (4) (main manuscript).

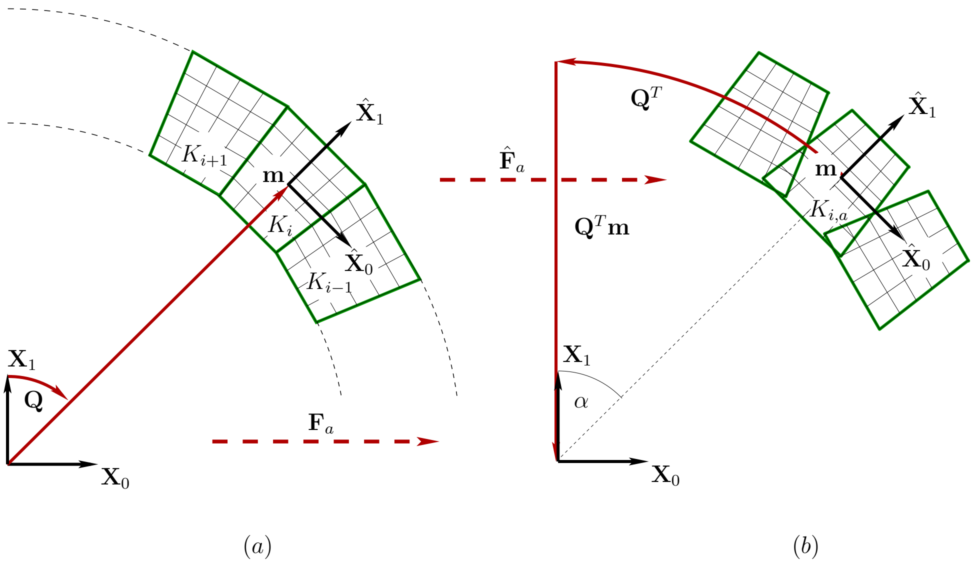


**Figure A.** (a) Transformation from the reference system $\mathbf{X}$ to the local coordinate system $\hat{\mathbf{X}}$ located in the centroid $\mathbf{m}$ of an arbitrary biological cell $K_i$ (green) by a rotation $\mathbf{Q}$ and a translation by $\mathbf{m}$. (b) The inverse transformation from $\hat{\mathbf{X}}_a$ to $\hat{\mathbf{X}}$ by $\mathbf{Q}^T$ and the translation $-\mathbf{Q}^T\mathbf{m}$.

For apical and basal constriction, the active deformation gradient is given by $\mathbf{F}_a = \mathbf{Q}^T \hat{\mathbf{F}}_a \mathbf{Q}$, where $\hat{\mathbf{F}}_a(C)$ depends on the local morphogen concentration and $\mathbf{Q}$ is a rotation matrix, see Eq. (6) of the main manuscript. As presented there, each biological cell constricts individually but in the same manner. The idea is now to prescribe the same active deformation for each cell and transform that tensor such that its orientation coincides with the orientation for the specific biological cell under consideration. Therefore, we have introduced local coordinate systems $\hat{\mathbf{X}}$ in the origin $\mathbf{m}$ of every biological cell such that $\hat{\mathbf{F}}_a$ is identical in all these systems. The whole active deformation tensor $\mathbf{F}_a$ is now given as follows: The a rotation $\mathbf{Q}_i = \mathbf{Q}|_{K_i}$ to the system $\hat{\mathbf{X}}_i$ of the considered biological cell $K_i$, the application of the constriction $\hat{\mathbf{F}}_a$ (independent of $i$ i.e. of the current cell) and the backwards rotation $\mathbf{Q}_i^T$ and the translation $-\mathbf{Q}_i^T \mathbf{m}_i$ of the argument. We have illustrated these mappings in Fig. A with the restriction to two dimensions to simplify the presentation. These mappings are easily extended to three dimensions defining $\mathbf{Q}$ as two rotations around coordinate axes.

Note that both rotations $\mathbf{Q}$ and $\mathbf{R}$, introduced in this section, are very similar. On the one hand however, $\mathbf{Q}$ depends on the midpoint $\mathbf{m}_i$ of the biological cell $K_i$ under consideration, i.e. $\mathbf{Q}|_{K_i} = \mathbf{Q}_i(\mathbf{m}_i)$, and is constant within cells. On the other, $\mathbf{R}(\mathbf{X})$ depends on each point $\mathbf{X}$.

# Derivation of mechanical invariants

A tensor $\mathbf{A}$ in three dimensions posseses three so-called principal scalar invarants $I_i$, $i = 1, 2, 3$. The first invariant of a tensor $\mathbf{A}$ is always the trace and last invariant is its determinant. In three dimensions, this gives the tensor invariants

$$
\begin{aligned}
I_1(\mathbf{A}) &= \mathrm{tr}(\mathbf{A}) = \sum_i A_{ii}, \\
I_2(\mathbf{A}) &= \frac{1}{2}(tr(\mathbf{A})^2 - tr(\mathbf{A}^2)) \\
&= A_{00}A_{11} + A_{11}A_{22} + A_{00}A_{22} - A_{01}A_{10} - A_{12}A_{21} - A_{02}A_{20} \\
I_3(\mathbf{A}) &= \det(\mathbf{A}) = \sum_{i,j,k} \epsilon_{ijk} A_{1i} A_{2,j} A_{3,k},
\end{aligned}
$$

where $\epsilon_{ijk}$ denotes the Levi-Civita symbol. Consequently, if our mechanical feedback influencing the production of morphogen is merely depending on these invariants, it does not change with the rotation of the coordinate system. To be more precise, the coupling term

$$R = R(I_1(\mathbf{A}), I_2(\mathbf{A}), I_3(\mathbf{A}), C)$$

will be objective for an objective tensor $\mathbf{A}$. Obviously, it is crucial that physical quantities such as deformation, stress or strain are invariant relative to a change of observer which should not effect the properties of the material under consideration. For our purpose, it is thus reasonable to take the tensor invariants of these quantities as a mechanical feedback in our coupling $R$. For a better understanding of objectivity, we refer to the presention in section 5.2 in Holzapfel [1].

Clearly, the second Piola-Kirchhoff stress tensor $\mathbf{\Sigma}$ is intrinsically objective, since it is parameterized by material coordinates only. Furthermore, it can be shown that the first Piola-Kirchhoff stress tensor $\mathbf{P}$ and the deformation gradient $\mathbf{F}$ are objective, although they behave like vectors. However, one of their indices is described in the reference configuration, which makes them objective nonetheless. Simple argumentation shows that the Cauchy stress tensor $\boldsymbol{\sigma}$ and the Green-Lagrange strain tensor $\mathbf{E}$ are objective as well.

This gives us several potential candidates for the mechanical feedback. Since we are in the reference configuration, it is natural to choose the second Piola-Kirchhoff stress tensor $\mathbf{\Sigma}$ form the available stress measures. With regard to extensive numerical testing, we restrict ourselves to the determinant of the deformation gradient $I_3(\mathbf{F}) = \det(\mathbf{F})$ for the purpose of this publication, see Eq. (4) of the main manuscript. Note, that different feedback loops also showed promising results which are intended to be published in futur works.

# Robustness of pattern formation

We have demonsrated that our simple, positive feedback loops lead to mechanochemical pattern formation (see Fig. 2 from the main manuscript for basal constriction and Fig. 3 main manuscript for apical constriction). In the following, we focus on the robustness of such patterns with respect to changes in the initial conditions, the model geometry and the model parameters/assumptions.

## Robustness with regard to initial conditions

Firstly, we demonstrate, that our positive feedback loop produces the same number and quality of patterns if we change the initial morphogen distribution. Regarding pattern formation for basal constriction

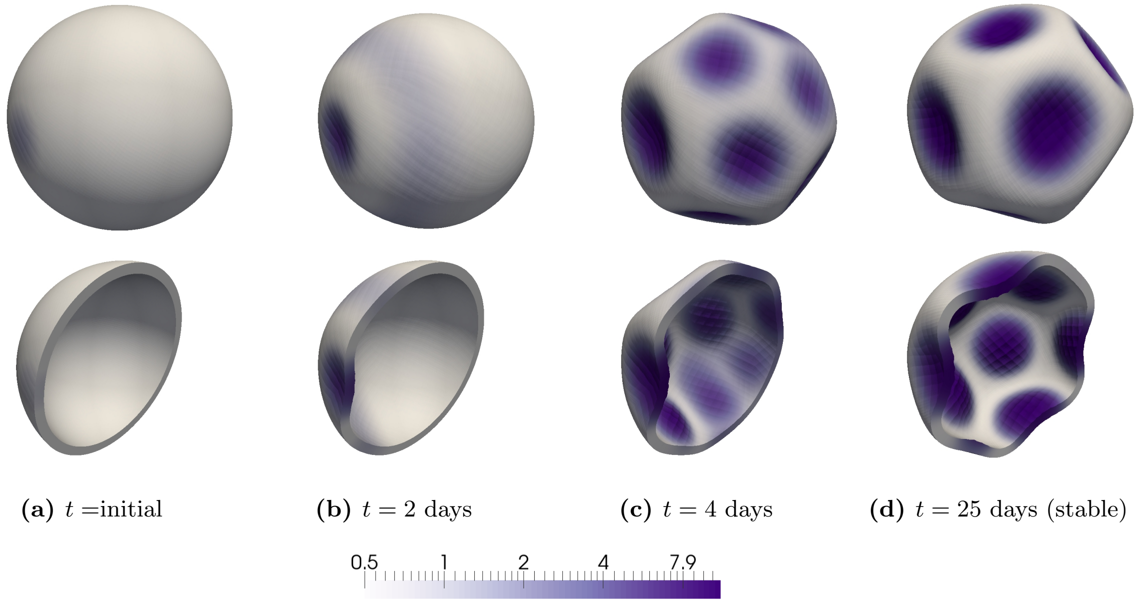


**(a)** $t$ =initial **(b)** $t = 2$ days **(c)** $t = 4$ days **(d)** $t = 25$ days (stable)

**Figure B.** (a)-(d) Simulation snapshots showing spontaneous pattern formation based on a simple mechanochemical feedback loop including basal constriction. Here, morphogen is initially distributed with a spot at one side of the sphere. In the lower row, the 3D tissue body has been sliced just for the purpose of a better visualisation.

(c.f. Fig. 2 main manuscript) we now start with just one weak morphogen spot at the side of the sphere (instead of a stochastic morphogen distribution) and again obtain a stationary solution with the same number and size of mechanochemical patterns (Fig. B) where the solution still slightly changes after four days but is stationary after 25 days.
Secondly, the same robustness with regard to a change in the initial conditions is observed for apical constriction: also here, starting with a weak morphogen spot at the side of the sphere (instead of a stochastic distribution) our feedback mechanism again results in the same number and quality of transient patterns and finally also in a gastrulation event with an annulus of high concentration around the invagination (please compare Fig. 3 from the main document and Fig. C).

## Comparable patterns for apical and basal constricton

At fist sight, from Fig. 2-3 in the main manuscript, it might not be obvious that the mechanochemical patterns gained by our positive feedback loops for apical and basal constriction are actually strongly related: In Fig. D we compare simulation snapshots for both cases with an identical orientation of the coordinate system and at the identical time. Apparently, the curvature patterns are very similar where the chemical concentrations just are negatively correlated.

Yet, only for apical constriction it was possible to simulate a complete gastrulation event, c.f. Fig. 3 (main manuscript) and Fig. C. In general, these events seem to occur in both considered feedback loops since there is usually one dominating curvature/morphogen patch which evolve faster than others, for instance see Fig. 3 (main manuscript) after four days (apical constriction) or Fig. B after two days

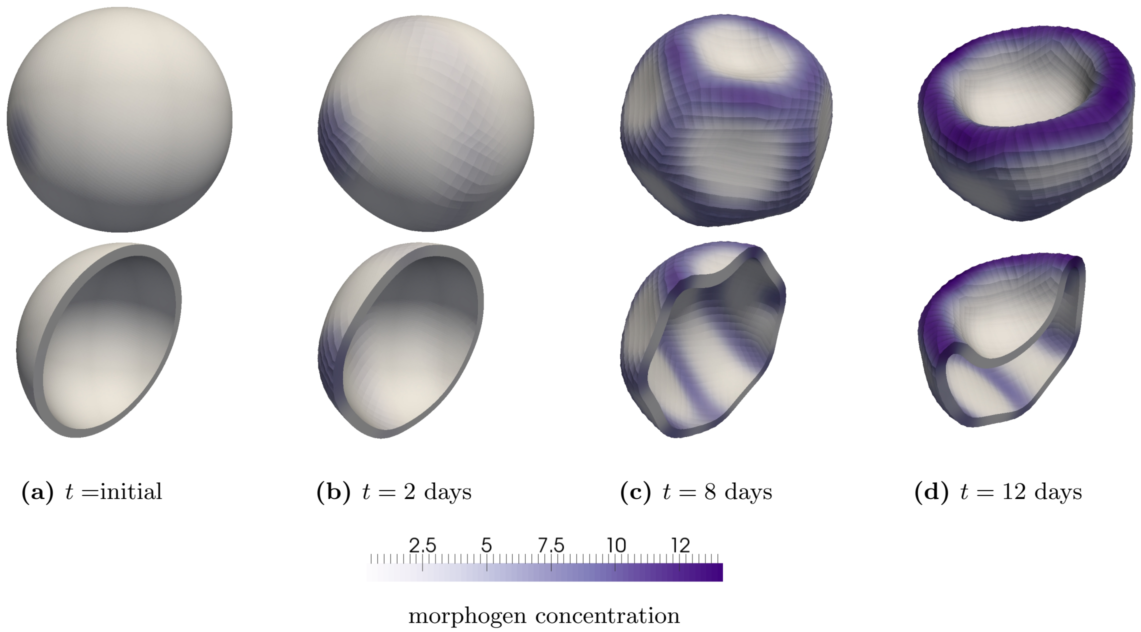


**Figure C.** (a)-(d) Simulation snapshots showing spontaneous pattern formation based on a simple mechanochemical feedback loop including apical constriction. Here, morphogen is initially distributed with a spot at one side of the sphere. In the lower row, the 3D tissue body has been sliced just for the purpose of a better visualisation.

(basal constriction). Note that this statement neglects artificial configurations, e.g. of two identical, opposing initial morphogen circles for apical constriction and presumes that mechanochemical coupling is strong enough to produce large patterns. However, calculations for basal constriction break down at an earlier stage, i.e., Newton's method no longer converges, for instance, if we increase the morphogen production rate to obtain larger deformations and an eventual gastrulation rather than the stationary patterns observed in Fig. 2 (main manuscript). However, some of our calculations indicate that a mechanical feedback relying on stress might produce more stable gastrulation patterns (results not shown).

This gastrulation event for our feedback loop based on apical constriction means a significant topological change of the tissue geometry: eventually, we do not any longer observe a further increase in morphogen concentration but the invagination continues until the model breaks down (as discussed in the main manuscript). However, first simulations regarding an internal volume constraint are very promising: if we prescribe a surface force in normal, outward direction of the internal boundary of the tissue sphere which becomes stronger if internal volume is lost, this pressure stops the gastrulation event and seems to ensure a stationary result (c.f. Fig. F (c) ).

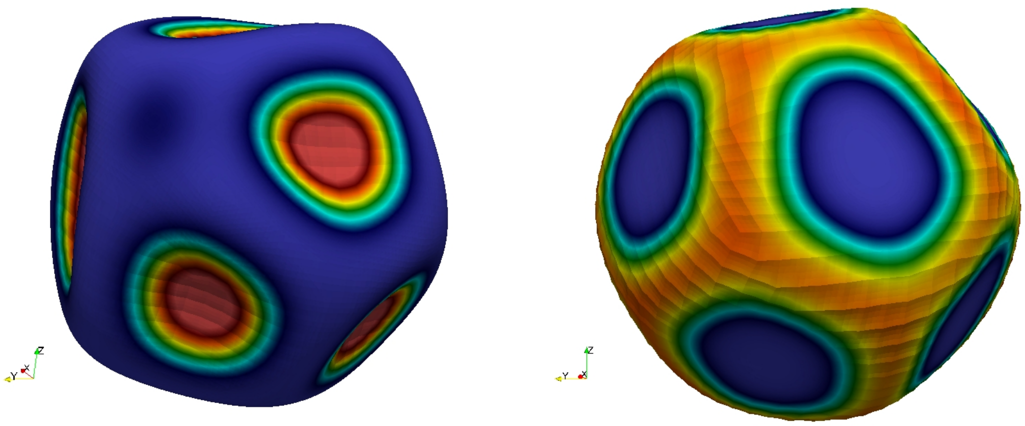


**(a)** Basal constriction, $t = 4$ days **(b)** Apical constriction, $t = 4$ days

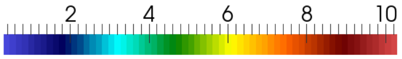


morphogen concentration

**Figure D.** Simulation snapshots showing pattern formation driven by a simple feedback loop based on basal (a) and apical (b) constriction, respectively. Both snapshots have the same coordinate orientation. The new color map (compared to the main manusrcipt) was used for a better illustration of the similarities between both results.

## Robustness regarding the model geometry and parameters

As described above, changes in the initial condition lead to qualitatively and quantitatively comparable patterns. In contrast, changes in model parameters or geometry may also influence the number, regularity or size of patterns. One example is given by the tangential diffusion, which appears to smooth/regularise morphogen patterns: simulation results show that quartering the tangential diffusion leads to more but smaller patterns (Fig. E (a)). In fact, tangential diffusion is not essential to produce patterns at all: Without any tangential diffusion we still obtain mechanochemical patterns, shown for basal constriction in Fig. F (a) (while this configuration obviously contains the modelling error that morphogen also no longer diffuses in tangential directions within biological cells). At first, these patterns are very tiny but as they deform surrounding tissue which is stretched, this stretch leads to morphogen production via our positive feedback loop and the patterns slowly extend (tangential diffusion also accelerates pattern formation).

We also investigated the influence of the model geometry on resulting patterns. Firstly, we considered a smaller tissue sphere consisting of only 384 biological cells (see Fig. E (b)). It appears that we obtain comparable patterns to those of our original domain in Fig. 2 (main manuscript) and Fig B, however there are less morphogen patches due to the smaller surface of this sphere.

Additionally, we observe that morphogen/curvature patches dominate larger parts of the tissue sphere if the tissue thickness is decreased (Fig E (c)). Contrary, there are fewer patches deforming larger parts of the tissue if the thickness of the sphere is increased, c.f. Fig E (d) (which in principle also holds for Fig E(b) where the relative tissue thickness is increased in comparison to our usual domain).

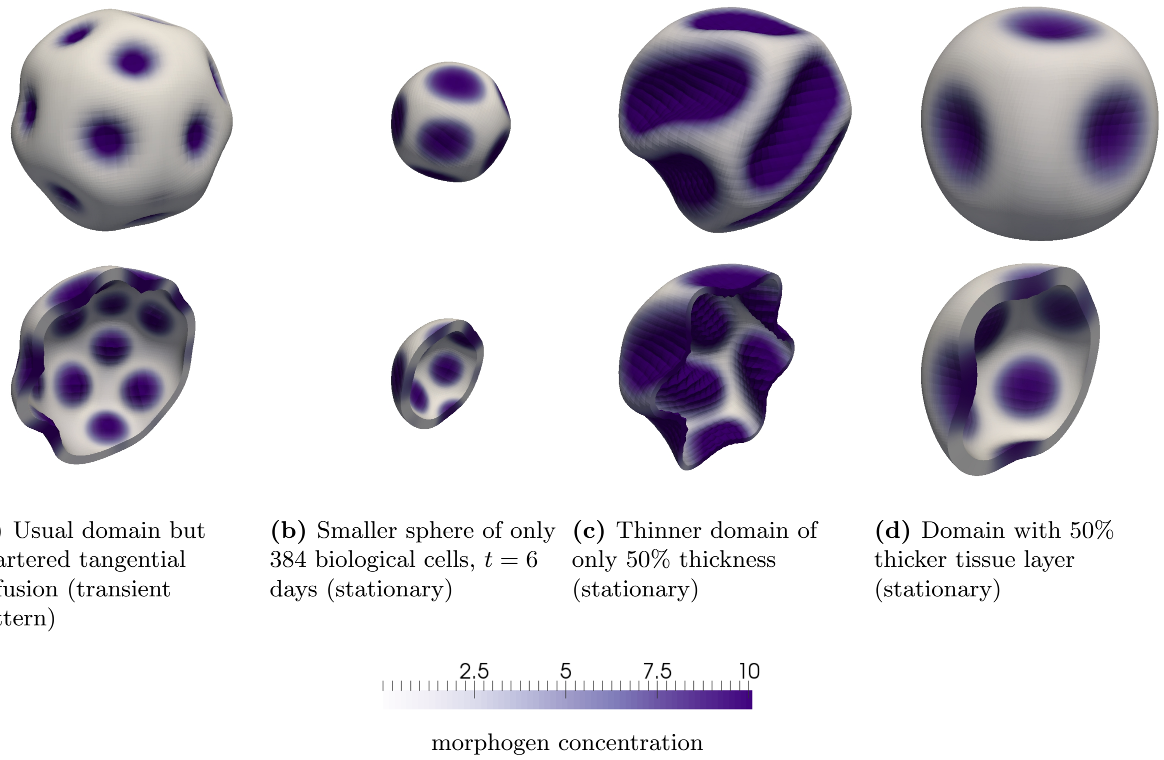


**(a)** Usual domain but quartered tangential diffusion (transient pattern)

**(b)** Smaller sphere of only 384 biological cells, $t = 6$ days (stationary)

**(c)** Thinner domain of only 50% thickness (stationary)

**(d)** Domain with 50% thicker tissue layer (stationary)

**Figure E.** (a)-(d) Simulation snapshots showing resulting patterns based on changes in lateral diffusion, system size, and tissue thickness. In the lower row, the 3D tissue body has been sliced just for the purpose of a better visualisation.

In the "parameter setup" section of the main manuscript we stated that we essentially balance tangential diffusion and the morphogen production rate $k_2$ to obtain mechanochemical patterns. Whereas the effect of tangential diffusion was just discussed, the influence of the latter can be illustrated as follows. A larger production rate results in higher morphogen concentrations which in turn induce larger active cell-shape changes i.e. larger deformations. Analog to patterns in a thicker tissue sphere, these patterns dominate larger parts of the domain. For instance, we usually expect more patterns on the thinner domain in Fig E (c) which is outbalanced by the higher morphogen concentrations and thus larger deformations in this example.

In summary, we see, that smaller lateral diffusion and thinner domains or lower morphogen production result in more patterns; larger diffusion, thicker domains and higher morphogen levels lead in contrast to fewer and larger patterns.

## Robustness regarding active deformations

Finally, we show that our simple feedback loops also lead to mechanochemical patterns if we change the nature of the active deformations. If we e.g. redefine the active deformation tensor such that it constricts one side of the cell without actively expanding the other side (i.e., the active deformation is not any longer volume preserving) we still obtain regular patterns (Fig. F (b)). Interestingly, morphogen patches are less spherical but rather develop to "double-patches", i.e., two superimposed patches constituting

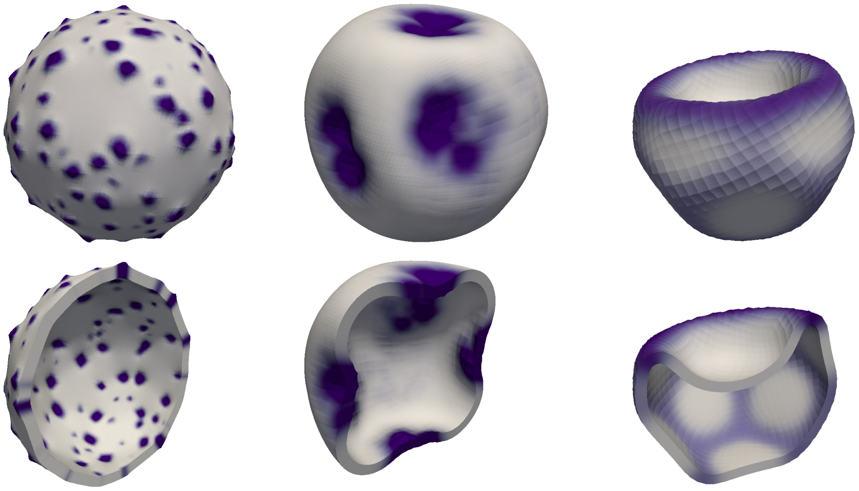

**(a)** Apical constriction without any tangential diffusion (transient pattern)

**(b)** Basal constriction only in the outer half of biological cells (transient pattern)

**(c)** Apical constriction including an internal volume constraint (transient pattern)

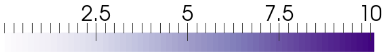


morphogen concentration

**Figure F.** (a)-(c) Simulation snapshots showing spontaneous pattern formation based on a simple mechanochemical feedback loop including apical (a) and basal (b)+(c) constriction. Here, we investigate the robustness of resulting patterns with respect to changes in lateral morphogen diffusion respectively non-volume preserving active deformations. (d) Simulation snapshots showing the gastrulation based apical constriction, which was stopped by an internal volume constraint. In the lower row, the 3D tissue body has been sliced just for the purpose of a better visualisation.

together a dumbbell-shape.

# References

1. Holzapfel GA (2010) Nonlinear Solid Mechanics - A continuum approach for engineering. John Wiley & Sons, LTD.

2. Ciarlet P (1997) Mathematical elasticity, Vol II: Theory of Plates, Series "Studies in Mathematics and its Applications". North Holland, Amsterdam.

3. Lubarda V, Hoger A (2002) On the mechanics of solids with a growing mass. International Journal of Solids and Structures 39: 4627-4664.

4. Richter T (2017) Fluid-structure Interactions. Models, Analysis and Finite Elements., volume 118 of *Lecture notes in computational science and engineering.* Springer.
